# A demonstration of cone function plasticity after gene therapy in achromatopsia

**DOI:** 10.1093/brain/awac226

**Published:** 2022-08-24

**Authors:** Mahtab Farahbakhsh, Elaine J Anderson, Roni O Maimon-Mor, Andy Rider, John A Greenwood, Nashila Hirji, Serena Zaman, Pete R Jones, D Samuel Schwarzkopf, Geraint Rees, Michel Michaelides, Tessa M Dekker

**Affiliations:** UCL Institute of Ophthalmology, University College London, London EC1V 9EL, UK; Experimental Psychology, University College London, London WC1H 0AP, UK; UCL Institute of Ophthalmology, University College London, London EC1V 9EL, UK; UCL Institute of Cognitive Neuroscience, University College London, London WC1N 3AZ, UK; The Wellcome Centre for Human Neuroimaging, University College London, London WC1N 3AR, UK; UCL Institute of Ophthalmology, University College London, London EC1V 9EL, UK; Experimental Psychology, University College London, London WC1H 0AP, UK; UCL Institute of Ophthalmology, University College London, London EC1V 9EL, UK; Experimental Psychology, University College London, London WC1H 0AP, UK; UCL Institute of Ophthalmology, University College London, London EC1V 9EL, UK; Moorfields Eye Hospital, London EC1V 2PD, UK; UCL Institute of Ophthalmology, University College London, London EC1V 9EL, UK; Moorfields Eye Hospital, London EC1V 2PD, UK; UCL Institute of Ophthalmology, University College London, London EC1V 9EL, UK; Division of Optometry and Visual Sciences; School of Health Sciences; City, University of London, London EC1V 0HB, UK; Experimental Psychology, University College London, London WC1H 0AP, UK; School of Optometry and Vision Science, University of Auckland, Auckland 1023, New Zealand; UCL Institute of Cognitive Neuroscience, University College London, London WC1N 3AZ, UK; The Wellcome Centre for Human Neuroimaging, University College London, London WC1N 3AR, UK; UCL Institute of Ophthalmology, University College London, London EC1V 9EL, UK; Moorfields Eye Hospital, London EC1V 2PD, UK; UCL Institute of Ophthalmology, University College London, London EC1V 9EL, UK; Experimental Psychology, University College London, London WC1H 0AP, UK

**Keywords:** gene therapy, photoreceptors, achromatopsia, fMRI, visual cortex

## Abstract

Recent advances in regenerative therapy have placed the treatment of previously incurable eye diseases within arms’ reach. Achromatopsia is a severe monogenic heritable retinal disease that disrupts cone function from birth, leaving patients with complete colour blindness, low acuity, photosensitivity and nystagmus. While successful gene-replacement therapy in non-primate models of achromatopsia has raised widespread hopes for clinical treatment, it was yet to be determined if and how these therapies can induce new cone function in the human brain. Using a novel multimodal approach, we demonstrate for the first time that gene therapy can successfully activate dormant cone-mediated pathways in children with achromatopsia (CNGA3- and CNGB3-associated, 10–15 years). To test this, we combined functional MRI population receptive field mapping and psychophysics with stimuli that selectively measure cone photoreceptor signalling. We measured cortical and visual cone function before and after gene therapy in four paediatric patients, evaluating treatment-related change against benchmark data from untreated patients (*n* = 9) and normal-sighted participants (*n* = 28). After treatment, two of the four children displayed strong evidence for novel cone-mediated signals in visual cortex, with a retinotopic pattern that was not present in untreated achromatopsia and which is highly unlikely to emerge by chance. Importantly, this change was paired with a significant improvement in psychophysical measures of cone-mediated visual function. These improvements were specific to the treated eye, and provide strong evidence for successful read-out and use of new cone-mediated information. These data show for the first time that gene replacement therapy in achromatopsia within the plastic period of development can awaken dormant cone-signalling pathways after years of deprivation. This reveals unprecedented neural plasticity in the developing human nervous system and offers great promise for emerging regenerative therapies.

## Teaser

Using fMRI and psychophysics, Farahbakhsh *et al.* show that gene therapy can successfully activate dormant cone photoreceptor pathways in children with achromatopsia, a monogenic disease that disrupts cone function and causes complete loss of colour vision. The changes are paired with improvements in visual function.

## Introduction

Achromatopsia (ACHM) is a non-progressive recessively inherited retinal disorder in which disease-causing sequence variants in a single gene prevent cone photoreceptors from signalling. ACHM occurs in ∼1:30 000 births,^1,2^ with the most prevalent variants located in two genes, *CNGA3* (∼30% of European and US cases) and *CNGB3* (∼50% of cases).^3^ These genes encode the α and β subunits of the cone cyclic nucleotide-gated (CNG) channel, respectively, both essential for the cone phototransduction cascade. As a result, vision in patients with ACHM is rod-dominated, and characterized by low acuity (6/36–6/60), insensitivity to chromatic contrast (complete colour blindness), day-blindness, photophobia and involuntary oscillation of the eyes (pendular nystagmus).^4^

The retinal integrity of the two commonest forms of ACHM (mutations in *CNGA3* and *CNGB3*) have been studied in great detail (cross-sectionally and longitudinally), both with high-resolution optical coherence tomography to investigate retinal lamination and also cellular imaging to directly probe the photoreceptor mosaic *in vivo*.^4–7^ These studies have identified that although there is a marked reduction in cone cell density, all patients have residual cone cells that could be targeted for rescue, albeit with significant intersubject variability in number.

ACHM is a promising candidate for genetic therapy, given its well understood genetic aetiology, availability of animal models, the presence of potentially viable cone cells and the accessibility and low immune response of the retina to surgical intervention. The feasibility of using gene therapy safely to successfully treat inherited eye disease was demonstrated recently with the first FDA- and EMA-approved gene therapy for RPE65-associated retinal dystrophy, Leber’s congenital amaurosis, a severe early-onset blinding disease.^8^ There are currently three phase I/II gene therapy trials for *CNGA3*-associated ACHM (NCT03758404, NCT02935517 and NCT02610582), and two phase I/II gene therapy trials for *CNGB3*-ACHM (NCT03001310 and NCT02599922).

Recently, two clinical trials published the results of subretinal gene therapy applied to nine (NCT02610582) and two (NCT02935517) treated adults with *CNGA3*-associated ACHM.^9,10^ These studies tested for change on measures of visual acuity, photophobia, contrast sensitivity, flicker fusion, colour thresholds and visual cortex function after gene therapy, and reported modest improvements in function in the treated eye compared to the untreated eye for some measures in some patients. One reason for these modest effects may be that for the mature visual system, functional benefits of gene therapy are limited by reduced retinocortical plasticity. In adult patients with ACHM there is evidence for altered structure and function of cortex that normally processes cone information.^11,12^ Detrimental effects of early visual deprivation have been shown to become more entrenched with age.^13,14^ Therefore, it is plausible that the cortical capacity for processing new cone signals becomes increasingly limited with advancing age. Indeed, in a mouse model of ACHM, gene therapy had greater functional benefit when applied in young animals.^15^ For these reasons, therapeutic benefits in ACHM might be enhanced or unlocked by exploiting the inherent plasticity of the developing brain. The current study therefore investigates, for the first time, the effects of gene therapy on visual cone function in children with ACHM.

For cone-mediated vision to be possible after treatment, cone signals restored in the retina must first be successfully transmitted to visual cortex. Moreover, to support functional vision the spatial tuning of these new cone-mediated responses must largely follow the canonical retinotopic map structure, because brain-wide visual processing relies on this spatial encoding scheme.^16^ Some coarse retinotopic connections to the cortex are likely to be retained even in the dormant cone photoreceptor system in ACHM, as these are neurochemically constrained before birth and preserved to some degree in nearly all known cases of atypical visual development, including congenital blindness.^17^ However, it has been suggested that the lifelong lack of cone-mediated signalling and enhanced competitive pressures from rod-dominated vision may alter these connections in ACHM patients and limit recovery.^12,18^ To assess the functional benefits of gene therapy in ACHM, it is therefore crucial to understand the degree to which the cortex can achieve normal spatial tuning when receiving input from successfully treated retinal cones.

To test this, we introduce a novel functional MRI (fMRI) mapping approach that separates emerging post-treatment cone signals from existing rod-driven signals in patients. Unlike previous studies on gene therapy in ACHM,^10^ this approach allows us to pinpoint any changes in visual function after treatment directly to the targeted cone photoreceptor system. Specifically, we use a ‘silent substitution’ technique to independently manipulate rod and cone signalling.^19^ We then use the resulting cone- and rod-selective stimuli to test for the first time not only whether gene therapy can induce new cone signalling from the retina to visual cortex, but also the degree to which neuronal retinotopic tuning profiles are preserved in these newly engaged pathways. We complement fMRI measures with objective psychophysical tests of cone contrast perception to validate our neuroimaging approach and, importantly, test if any newly observed cone signals are indeed utilized to improve visual function.

We used this approach to test for new cone function after gene therapy in four children with ACHM aged 10–15 years (CNGA3- or CNGB3-associated). Each child was enrolled in a phase 1/2 clinical trial investigating subretinal gene therapy with adeno-associated virus vectors expressing CNGA3 or CNGB3 (NCT03758404 and NCT03001310, see clinicaltrials.gov). The effects of gene therapy in these children are contextualized against large datasets from additional untreated patients with ACHM (*n* = 9) and normal-sighted control participants (*n* = 28) tested under identical circumstances. These groups allow us to evaluate the degree to which visual function after treatment goes above and beyond that normally seen in ACHM and the degree to which new cone vision is normalized.

Our results provide strong novel evidence that retinal gene therapy can successfully activate dormant cone photoreceptor pathways and evoke new visual function. For both the fMRI and psychophysical measures, two of the four treated patients with ACHM demonstrated therapy-induced improvement in cone function 6–14 months after treatment. Before treatment, these two patients showed no evidence of cone function on any of our tests, scoring within the range of untreated patients with ACHM. After treatment, their measures closely resembled those from normal-sighted controls with functioning cone pathways on converging behavioural and neural indices.

## Materials and methods

### Participants

We present data from four child patients (age range = 10–15 years) with genetically confirmed ACHM, before and after undergoing subretinal gene therapy with adeno-associated virus vectors expressing CNGA3 or CNGB3. Genetic testing confirmed that Patients Tr1, Tr2 and Tr4 had the *CNGA3* mutations and Patient Tr3 the *CNGB3* mutation. They were enrolled in clinical trials NCT03758404 and NCT03001310. These patients were tested once before and again 6–14 months after gene therapy was applied to the eye with lowest acuity (Table 1). Identical measures were collected from two comparison groups. One group comprised seven additional children (11 paediatric patients in total: mean age = 11.27, range = 8–15 years, SD = 2.49) and two adults in their twenties with genetically confirmed *CNGA3* or *CNGB3*-associated ACHM (Table 1). The second control group comprised 28 normal-sighted controls (16 children: mean age = 11.10, range = 6–15 years, SD = 2.62; 12 adults: mean age = 25.14, range = 19–34 years, SD = 4.53). Some participants had incomplete datasets: for Patient Utr2 (Table 1), no psychophysics data were collected, and for Patients Utr6 and Tr2, the ridge test was not collected. For two normal-sighted children the rod-mediated population receptive field (pRF) map was not collected. All participants met MRI safety inclusion criteria and had no other known neurological disorders. Data collection had ethics approval (separate from clinical trials) from the national ethics committee for patients (REC reference: 12/LO/1196; IRAS code: 106506) and the UCL ethics committee for normal-sighted control participants (#4846/001).

**Table 1:** Participant details including clinical visual acuity measures, psychophysical thresholds and fMRI data measures regarding the correspondence and correlation between the cone- and rod-driven cortical maps

Age group	Genotype	Visual acuity BCVA^a^ (LogMAR)			Cone sensitivity (threshold)		fMRI				pRF size^d^	Post-treatment test^e^
		Left eye	Right eye	Both	Square	Ridge	Intercept	Slope	AIC_W_^b^	CC_FL_^c^		
Pre-treatment treated patients with ACHM													
**Tr1**	**Child**	**CNGA3**	**0**.**94**^f^	**0**.**92**	**0**.**86**	**0**.**24**	**0**.**30**	**125**.**25**	**−0**.**14**	**0**	**−0**.**05**	**1**.**69**	**6**
**Tr2**	**Child**	**CNGA3**	**0**.**76**	**0**.**80**^f^	**0**.**70**	**0**.**46**	**N/A**	**32**.**29**	**0**.**02**	**0**	**−0**.**03**	**1**.**91**	**8**
Tr3	Child	CNGB3	0.80	0.84^f^	0.84	0.34	0.34	49.36	0.11	0	−0.03	1.68	14
Tr4	Child	CNGA3	0.78^f^	0.76	0.78	0.34	0.38	33.15	0.17	0	0.12	1.45	14
Post-treatment treated patients with ACHM													
**Tr1**	**Child**	**CNGA3**	**0**.**92**^f^	**0**.**84**	**0**.**82**	**0**.**16**	**0**.**16**	**−16**.**74**	**0**.**97**	**1**	**0**.**32**	**1**.**4**	
**Tr2**	**Child**	**CNGA3**	**0**.**76**	**0**.**86**^f^	**0**.**72**	**0**.**16**	**0**.**16**	**−8**.**27**	**0**.**82**	**1**	**0**.**60**	**1**.**76**	
Tr3	Child	CNGB3	0.56	0.72^f^	0.54	0.34	0.34	32.04	−0.12	0	−0.11	1.47	
Tr4	Child	CNGA3	0.78^f^	0.72	0.70	0.34	0.34	−2.00	−0.06	0	0.02	1.31	
Untreated patients with ACHM													
Utr1	Child	CNGB3	1.04	1.00	0.94	0.34	0.46	43.78	0.29	0	−0.31	2.27	
Utr2	Child	CNGB3	0.96	0.94	0.82	N/A	N/A	129.82	−0.08	0	−0.75	1.79	
Utr3	Child	CNGB3	0.84	0.94	0.82	0.42	0.38	−422.50	−17.36	0	−0.03	1.86	
Utr4	Child	CNGB3	0.90	0.90	0.86	0.34	0.30	85.97	0.29	0	0.70	1.46	
Utr5	Child	CNGB3	0.86	0.96	0.90	0.52	0.38	32.74	0.50	0	0.12	1.53	
Utr6	Child	CNGA3	0.86	0.92	0.92	0.46	N/A	−35.42	−4.64	0	0.12	2.3	
Utr7	Child	CNGA3	0.76	0.72	0.72	0.38	0.30	64.89	2.02	0	0.11	1.2	
Utr8	Adult	CNGA3	0.70	0.70	0.70	0.52	0.34	−3 × 10^6^	−2 × 10^4^	0	0.41	1.66	
Utr9	Adult	CNGA3	0.90	0.80	0.84	0.46	0.38	−97.29	0.50	0	−0.03	1.42	
Normal-sighted controls													
C1	Child	Normal^g^	N/A	N/A	0.02	0.16	0.16	2.20	1	1	0.86	1.92	
C2	Child	Normal^g^	N/A	N/A	−0.04	0.16	0.16	−4.97	1	1	0.74	1.56	

N/A indicates missing data. Measures from Patients Tr1 and Tr2 are presented using bold text. AIC_W_ = Akaike iInformation Criterion Weight; CC_FL_ = Fisher–Lee correlation coefficient.

^a^ Best corrected visual acuity.

^b^ Akaike weight.

^c^ Fisher–Lee correlation coefficient.

^d^ Rod mean pRF size.

^e^ Duration between the pre- and post-treatment behavioural and fMRI measures (in months).

^f^ Treated eye.

^g^ Normal-sighted control with no ACHM.

### Exclusions

Participant numbers above exclude additional normal-sighted and ACHM patients tested but removed from the analyses due to excessive head movement (one control, one patient), equipment problems (six controls, two patients), falling asleep during scanning (one patient), or missing MRI measures (two patients).

### Apparatus

We used a Siemens Avanto 1.5 T MRI scanner with a 30-channel coil (a 32-channel coil customised to remove view obstructions) to acquire structural and functional MRI data. Stimuli were presented on an MR-compatible LCD display (BOLDscreen 24, Cambridge Research Systems Ltd; 51 × 32 cm; 1920 × 1200 pixels) viewed through a mirror in the scanner at 105 cm distance. Participants were lying supine in the scanner, with fixation stability recorded where possible via a mirror, with an Eyelink 1000 at the back of bore. Behavioural psychophysics was also performed in the scanner room, after scanning, under similar viewing circumstances as the functional MRI data collection. Hardware were controlled using custom MATLAB code (R2016b, MathWorks, Natick, MA, USA), via the Psychophysics Toolbox 3.^20–22^

### Cone-selective stimuli

To selectively measure cone-mediated signal processing in psychophysics and fMRI, we created pairs of colours (chromatic pairs) carefully calibrated to induce different levels of activation in cone photoreceptors but identical levels of activation in rods, thus making them indiscriminable to rod photoreceptors (silent substitution).^19,23^ Specifically, these chromatic pairs were designed to selectively activate L- and M-cones while keeping rod activation constant. To achieve this, we computed transformation matrices to convert changes in the LCD screen’s red, green and blue (RGB) channel voltages, into changes in L-cone, M-cone and rod photoreceptor stimulation, using the RGB spectral output and the standard observer sensitivity functions for rod and cone photoreceptors^24,25^ (spectral output was measured with a Spectrascan Spectroradiometer, PR-655, PhotoResearch Inc., corrected for distortions from the fMRI mirror and neutral density filters). This allowed us to calculate the change in RGB voltage required to independently increment or decrement L- and M-cone or rod photoreceptor activity by prespecified proportions with respect to a baseline RGB value (mid-grey).

With three colour channels (RGB) in our set-up, it is possible to silence two photoreceptor types. However, for the cone-selective stimulus, we only required to silence one type of photoreceptor (rods), allowing more freedom in the chosen colour directions. This allowed us to account for any imperfect matching of rod activation (e.g. due to light measurement error, variations in rod sensitivity or screen inhomogeneity), by keeping the blue voltage constant and only varying the red and green channels. Because rods are relatively more sensitive to the blue channel than the L- or M-cones are, this shifts stimulus variations towards longer wavelengths where rods are less sensitive and errors in rod equating are likely to be small. Neutral density filters were used to present these stimuli in the mesopic/low photopic light range (0.6–1.3 cd/m^2^ for psychophysics, maximum 0.8 cd/m^2^ for fMRI), to keep viewing tolerable for this photosensitive population. For cone-selective stimulus validation tests, see ‘Validating cone selective stimuli' in the Supplementary material.

### Rod-selective stimuli

Rod-selective stimuli, only used in fMRI, were obtained using a similar silent substitution approach. We generated a single chromatic pair that kept L- and M-cone contrast constant while inducing a contrast response in rods. Again, because it is only possible to simultaneously silence two photoreceptor types with three colour channels, the resulting stimulus was not controlled for S-cone contribution. To eradicate the S-cone response, we presented these stimuli at very low light level (maximum luminance: 0.02 cd/m^2^). However, we cannot fully exclude that functioning S-cone photoreceptors were still activated and contributed to the rod-mediated maps in individuals with S-cone function (i.e. normal-sighted and potentially patients after successful therapy; see ‘Validating rod selective stimuli' in the Supplementary material for details).

### MRI sequences

Functional T_2_*-weighted multiband 2D echo-planar images^26^ were collected using a multiband sequence with 2.3 mm isotropic voxels [repetition time (TR) = 1000 ms, echo time (TE) = 55 ms, volumes = 348, flip angle = 75°, MB acceleration factor = 4, bandwidth = 1628 Hz/Px]. A high-resolution structural scan was acquired (T_1_-weighted 3D MPRAGE, 1 mm^3^ voxel size, bandwidth = 190 Hz/Px, 176 partitions, partition TR = 2730, TR = 8.4 ms, TE = 3.57, effective T_1_ = 1000 ms, flip angle = 7°). A lower-resolution structural scan was also obtained in the same orientation as the multiband sequence to aid co-registration between functional and structural images.

### Population receptive field mapping functional MRI

Although the gene therapy was applied to one eye, fMRI measures were collected binocularly for all subjects, due to time constraints and to reduce eye movements (as nystagmus can be enhanced with monocular occlusion). Inside the scanner, patients were constantly monitored for any signs of discomfort or movement via cameras and a two-way intercom. Participants first practised lying still while watching a cartoon, which was paused if head movement occurred. In case of large movements during scanning, participants were reminded to stay still, and if needed their head was repositioned and data were recollected.

**Figure 1:**
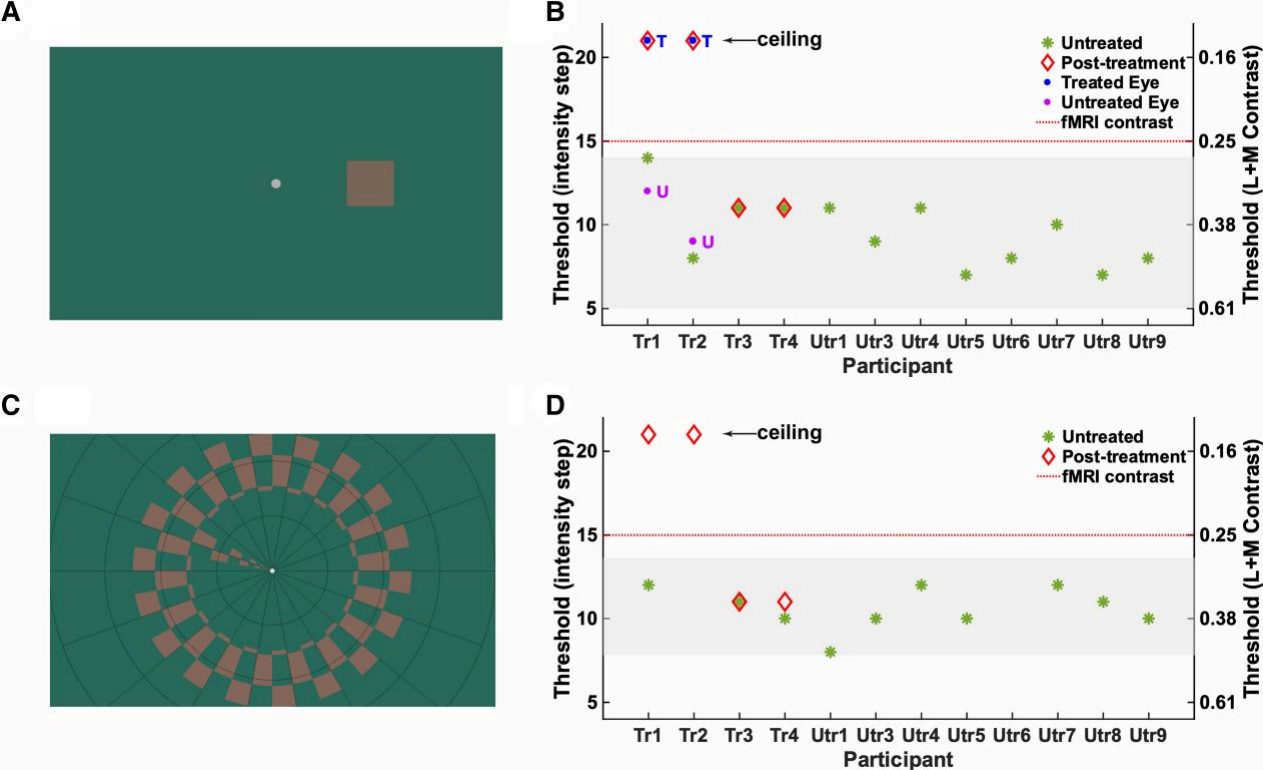
**Cone selective stimuli and psychophysics.** (**A**) Example of the lowest cone-selective contrast tested psychophysically, embedded in the 4AFC square localization psychophysics task. Participants judged the position of a target (size 3°), presented 6° to the left, right, above or below centre, with unlimited time to search and uncontrolled gaze. Note that stimulus appearance is screen-dependent. (**B**) Binocular contrast discrimination thresholds in the 4AFC square localization psychophysics task (50% correct) for children with ACHM. *Left y*-axis indicates contrast detection thresholds in units of staircase step with decreasing stimulus intensity (1 = highest contrast, 21 = lowest contrast), with the *right y*-axis indicating the corresponding L + M cone Michelson contrasts (see ‘Validating cone selective stimuli' in the Supplementary material for details). Green stars indicate measures for 12 untreated patients with ACHM. Shaded area: 95% prediction interval computed using the t-distribution quantile. Follow-up measures 6–14 months after treatment are shown for treated Patients Tr1–Tr4 (red diamonds). Post-treatment measures for Patients Tr1 and Tr2 were repeated monocularly for the treated eye (blue dots, ‘T’) and untreated eye (purple dot, ‘U’). (**C**) Cone-selective pRF mapping stimulus presented inside the scanner (at fixed contrast indicated by red dotted line in **B** and **D**), and in the Ridge motion discrimination task (maximum eccentricity 8.6°). In the scanner, participants detected target dimming at fixation. In the Ridge task they discriminated direction of ring movement (inward/outward). (**D**) As in **B** but showing binocular contrast discrimination thresholds (at 50% correct) for the Ridge motion discrimination task performed with the pRF mapping stimulus.

pRF mapping stimuli comprised a simultaneous rotating ring and contracting/expanding wedge, each made up of cone- or rod-selective chromatic pairs, embedded within a contrast-reversing checkerboard with 2 Hz reversal rate (Fig. 1B). Per run, the ring expanded/contracted for six cycles (48 s/cycle) with logarithmic eccentricity scaling.^27^ The wedge (20° angle) rotated clockwise/anticlockwise for eight cycles (36 s/cycle). Fixation baselines of 20 s were embedded at the start, at the mid-point and end of the run (total run duration 348 s). The stimuli covered a maximum eccentricity of 8.6°, and moved to a new position each 1 s TR.

The stimulus was overlaid with a small white central fixation dot (0.2° VA radius) and a black radial grid to encourage stable fixation. To keep participants engaged, they played a rewarded ‘kitten rescue mission game’ in which they pressed a button when detecting a fixation target luminance change. Fixation was monitored with a remote Eyelink 1000 Plus (SR Research, Ottawa, ON). Built-in gaze calibration was not possible in ACHM due to nystagmus so the camera was calibrated in advance on a healthy eye. Before every other run, patients fixated on a 5-point custom calibration, which allowed us to calibrate gaze measures *post hoc*^28^ (see ‘Eye movements' in the Supplementary material).

Each session had a fixed order consisting of: two runs with cone-selective checkerboards presented at low photopic light levels (maximum 0.8 cd/m^2^; Fig. 1B), a 15-min dark adaptation while participants listened to a story and structural scans were acquired, two runs with rod-selective checkerboards presented near scotopically (maximum 0.02 cd/m^2^; Supplementary Fig. 2A), two runs with non-selective checkerboards activating both rods and cones at mesopic luminance (maximum 0.5 cd/m^2^), used for region of interest selection.

### Behavioural psychophysics

After fMRI, cone contrast sensitivity was tested in patients with ACHM using two behavioural psychophysics tasks, with viewing conditions matched to those in the scanner. In each task, participants discriminated stimuli designed to selectively rely on cone function, consisting of the chromatic pairs described under ‘cone-selective stimuli’. For each chromatic pair, one RGB combination was used to define the target and the other to define the background. The cone contrast between the fore- and background decreased gradually from the brightest contrast using a 1-up/1-down procedure, converging on 50% correct. Before each task, we ensured that performance on practice trials with stimuli well above threshold was 100%. Cone contrast stimuli were embedded in two separate tasks: (i) a 4AFC Square Localization Psychophysics task; and (ii) a Ridge Motion Discrimination task.

#### 4AFC square localization psychophysics task

A 4AFC Square Localization Psychophysics task (henceforth Square Task) was used to assess cone contrast sensitivity objectively; participants were asked to locate a square (3° of visual angle), presented at 6° eccentricity to the left, right, above or below a central marker (0.4° VA; Fig. 1A) in a 4 Alternative Forced Choice (4AFC) task. Participants viewed the display binocularly, were not required to fixate and had unlimited time to search. Each 4AFC 1-up/1-down adaptive staircase continued until at least 14 reversals (mean *n* trials = 27) or >8 correct responses had occurred at the lowest cone contrast (ceiling). Due to time constraints, additional monocular measures were only obtained in select patients with significantly improved measures after treatment.

#### Ridge motion discrimination task

A Ridge Motion Discrimination task (henceforth Ridge Task) was used to test if these psychophysical threshold measures were representative of the stimulus in the scanner; we also measured binocular cone contrast sensitivity thresholds for the cone-selective ring-and-wedge (Fig. 1B). In this task, participants detected movement of a checkerboard ring (inward/outward) or wedge (clockwise/anticlockwise) reversing at 2 Hz. Both an incorrect response and a report that no checkerboard was visible after multiple replays triggered a step up in cone contrast, while a correct response triggered a step down in cone contrast (1-up/1-down adaptive staircase). Each staircase continued until at least eight reversals (mean *n* trials = 25) or >8 correct responses had occurred at the dimmest contrast level (ceiling).

### Data analysis

All functional data were pre-processed using SPM12 (http://www.fil.ion.ucl.ac.uk/spm). Functional volumes were realigned to the first image of each run to correct for head movement. All functional scans (collected pre- and post-treatment) were aligned to the high-resolution structural scan collected pre-treatment. For accuracy, a low-resolution structural image with the same orientation as the functional volumes was used as intermediate step to compute the co-registration matrix. FreeSurfer software (v5.3.0 with XQuartz v2.7.8, https://surfer.nmr.mgh.harvard.edu/) was used to construct 3D surface meshes for the right and left cortical hemispheres,^29^ using the recon-all pipeline. Any holes or edges were corrected manually using FreeSurfer Freeview tool. Preprocessed functional data were projected onto the surface using the MATLAB toolbox ‘SamSrf’ v5.84 (https://osf.io/2rgsm/) for further analyses.

To model population receptive fields, we used a symmetric bivariate Gaussian model, with mean (*x*, *y*) representing the preferred retinotopic location, and standard deviation (σ) representing pRF size. To identify the pRF model parameters (*x*, *y*, σ) that best predict the measured time series, a two-stage fitting procedure was employed. In a coarse fitting step, data were smoothed along the cortical surface [Gaussian kernel full-width at half-maximum (FWHM) = 5 mm], and a grid-search approach was used to identify model parameters that maximize the Pearson correlation between observed data and the pRF model’s predicted time course. Vertices with *R*^2^ > 0.05 were entered as starting value in a fine-fitting step, which used MATLAB’s fminsearch function to identify parameters that minimised the squared residual deviations between the model and unsmoothed data. Finally, best-fitting parameters were smoothed along the surface (FWHM = 3 mm), and *X* and *Y* position estimates were converted to eccentricity (distance from fixation) and polar angle.

pRFs with a low goodness of fit (*R*^2^) were excluded from all further analysis. As new cone-mediated pRF maps in ACHM after gene therapy were expected to have weaker signal (i.e. lower goodness of fit) than a normal cone-mediated map, we applied a relatively low statistical threshold of *R*^2^ > 0.03 corresponding to *P* = 0.0012. This allowed us to retain a representative proportion of ACHM post-treatment cone-map data (∼50% relative to no threshold). Importantly, results remained similar at a threshold of *R*^2^ > 0.05, which corresponds to a more stringent *P*-value of 0.00003 but only retains ∼20% of the ACHM cone map data (see ‘Statistical thresholds' in the Supplementary material for all results at a threshold of *R*^2^ > 0.05). Crucially, however, because our key index of cone-mediated cortical function tests for the presence of structured data (i.e. a retinotopic map), it is robust to lower *R*^2^ thresholds because more noise is unlikely to add more structure (see Supplementary material for simulations demonstrating this).

### Regions of interest

Visual regions were delineated manually on individual brains based on the non-selective pRF mapping stimulus. Polar-angle and eccentricity maps across all sessions were projected on an inflated cortical surface, and standard functional criteria were used to identify borders between V1, V2, and V3.^30–32^ For simplicity and comparison to other work,^10^ all pRF mapping results are averaged across areas V1–3 for this report.

### Functional MRI data quality control

Head and eye movement were minimized as much as possible during fMRI data collection (see ‘Population receptive field mapping functional MRI’ section). We also used stringent head-movement exclusion criteria and ensured that head movement and fixation stability were consistent between pre- and post-treatment scans within patients, and therefore unlikely to confound any treatment-related change in tests of cone function. The fact that retinotopic maps were obtained in Patients Tr1–4, untreated patients and normal-sighted controls with rod- and cone-selective stimuli under expected conditions shows that this approach provides sufficient data quality.

#### Head movement

To ensure high-quality data, we used standard motion exclusion criteria (excluding participants with >0.9 mm frame-wise displacement in >10% of volumes in any of the runs^33^). After applying these there were no differences in head displacement between the groups (see ‘Head movement' in the Supplementary material). We included one untreated patient (Utr1) despite exceeding this criterion in one cone-selective scan given the rarity of this group, and because main conclusions were not altered by removing this dataset. Crucially, all treated participants had very good head stability across all pre- and post-runs, so any changes in measures are unlikely to be driven by head movement (Supplementary Fig. 5B).

#### Fixation stability

As index of fixation stability, we calculated horizontal eye movement variability because it is the dominant direction of nystagmus in ACHM and because vertical eye movements are prone to blink, eyelash and scanner vibration artefacts (see ‘Head movement' in the Supplementary material). As expected, fixation stability in treated patients, both before and after treatment, was lower than in control participants. Crucially, however, in all treated patients, fixation stability in the cone-selective condition was well-matched to fixation in the rod-selective condition, for which retinotopic maps were obtained for each patient. Moreover, fixation stability remained consistent before and after treatment in Patients Tr1–4. Therefore, changes in measures are unlikely to be driven by eye movements.

### Combining child and adult data

We contextualize the effects of gene therapy on cone-mediated function in four children with ACHM against measures from untreated patients and normal-sighted controls. Given their rarity, our sample of untreated patients included both children and adults, which we matched in the normally sighted group. In our comparisons of these groups, we opted not to distinguish between data from children and adults. For normal-sighted controls, this was because we and others have shown that pRF tuning remains consistent from ages 6 to 8 years onwards in size, position, coverage, and shape.^34,35^ For untreated patients with ACHM, this was because the measures from two adults fell within the range of those of 13 children on all tests (Table 1), so treating all as one single group did not skew the results. This is consistent with the fact that neither children nor adults with ACHM have cone function before treatment, meaning that age differences are not expected in tests for presence or absence of a cortical cone map. It is possible that subtle differences in rod-mediated pRF tuning exist across child and adult patients and that these may predict opportunities for recovering dormant cone function, though we were unable to detect this with our small adult sample.

### Data availability

For GDPR and confidentiality reasons we are not able to make imaging data from paediatric participants publicly available. All stimulus presentation and data analysis code are available on request to the corresponding author.

## Results

### Behavioural psychophysics

We used behavioural psychophysics to test whether sensitivity to the contrast of cone-selective stimuli emerged after gene therapy in ACHM. To do so, we embedded chromatic stimuli designed to be indistinguishable to rods but to vary in the contrast they induce in L- and M-cones, in a 4AFC square localization psychophysics task (Fig. 1A). Cone contrast discrimination thresholds (expressed in average Michaelson Contrast for the L- and M-cones) were measured with a 1-up/1-down staircase. Note that normal-sighted individuals perform at ceiling with these stimuli, perceiving even the smallest cone contrast effortlessly.

With perfectly calibrated cone-selective stimuli, we would have expected patients with ACHM to not be able to detect any of these cone contrasts. However, Fig. 1C shows that the higher-contrast stimuli were perceived by this group: 12 untreated patients with ACHM obtained cone contrast sensitivity thresholds between 0.16 and 0.52. This was in line with stimulus validation tests (see ‘Validating cone selective stimuli' in the Supplementary material) showing that the light spectra emitted by high L- and M-cone contrast stimuli also induced some undesired contrasting activation in rod photoreceptors (i.e. they were not perfectly cone-selective), likely due to error in measurement and correction for the screen’s non-canonical gamma function. This made it crucial to establish a pre-treatment measure of the presumably rod photoreceptor-based limits of sensitivity to these stimuli. We considered an improvement beyond the untreated ACHM measures after treatment as evidence for a robust change in cone function.

After gene therapy, contrast sensitivity thresholds for Patients Tr1 and Tr2 had improved to ceiling level, exceeding both their pre-treatment performance and that of all other untreated patients. Thresholds of Patients Tr3 and Tr4 had remained unchanged. Moreover, the improvement in Patients Tr1 and Tr2 was specific to their treated eye; monocular cone contrast discrimination was at ceiling for the eye that had received gene therapy but remained at pre-treatment levels for the untreated eye (blue and purple dots in Fig. 1B). An improvement in best corrected visual acuity was not observed, despite the striking improvement in cone contrast detection in Patients Tr1 and Tr2 (Table 1).

To confirm that the cone-selective chromatic stimulus pair used in the scanner (contrast level indicated by red dotted line in Fig. 1B and D) was invisible to rod photoreceptors, we also embedded chromatic pairs in a 1-up/1-down ridge motion discrimination task using the ring/wedge pRF mapping stimulus (Fig. 1C). Binocular measures from this task corresponded well with the more rigorous measures from the 4AFC square localization psychophysics task (‘Validating cone selective stimuli' in the Supplementary material) and provided an independent replication of the treatment results. Crucially, the cone-selective contrast used for pRF mapping could not be detected by any untreated patient on either test (i.e. all thresholds in Fig. 1B and D fall below the red dotted line). Together with stimulus validation measures (Supplementary material), this confirms that this stimulus was accurately calibrated to selectively stimulate the cone pathway.

### Cone-mediated retinotopic map structure in visual cortex

**Figure 2:**
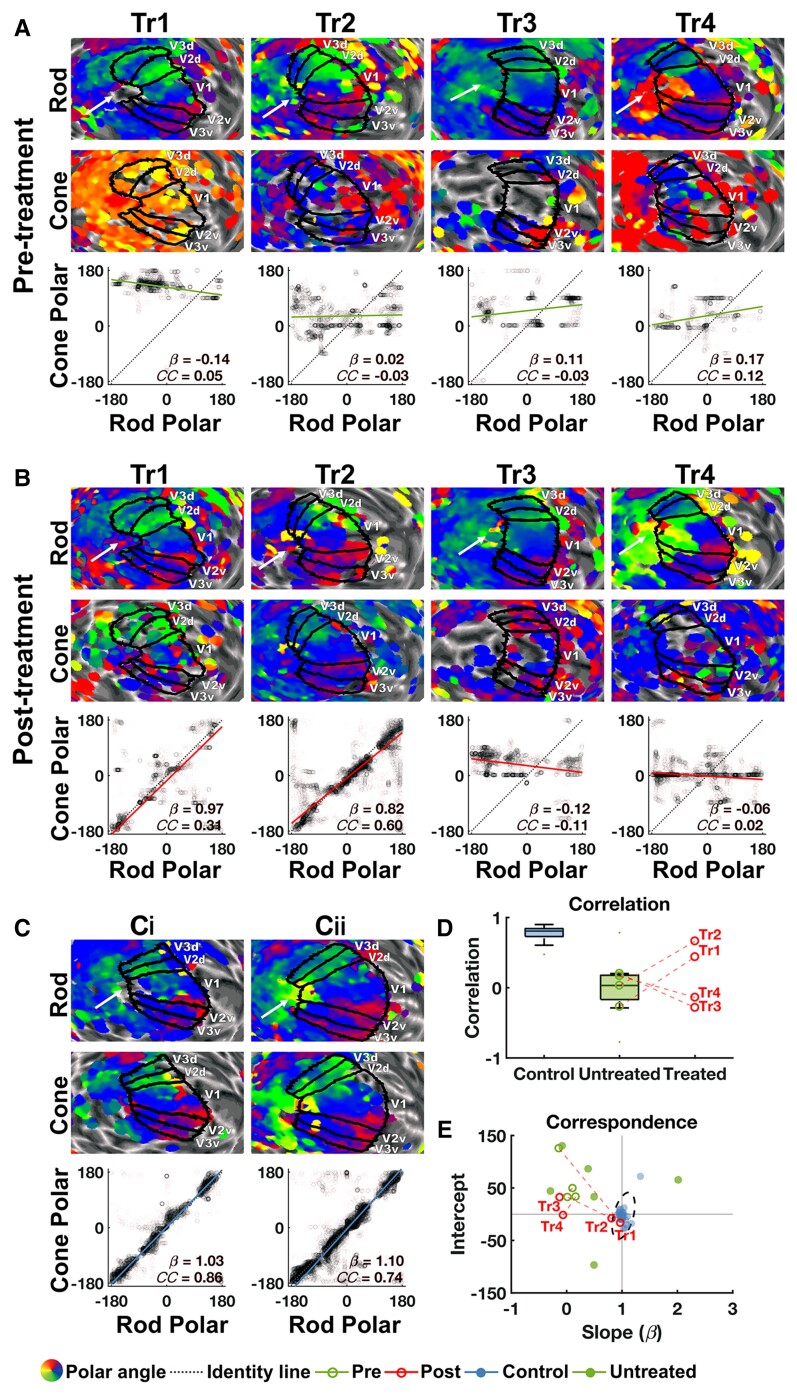
**Cone-mediated retinotopy in visual cortex.** Cone-mediated versus rod-mediated polar angle measures in areas V1–3 in four treated patients (Patients Tr1–4) before (**A**) and after (**B**) gene therapy and in two age-matched normal-sighted controls (**C**). For polar angle maps in (**A**–**C**), rod- and cone-mediated estimates were projected onto the left hemisphere cortical surface, inflated to a sphere and overlaid with individual V1–3 labels (maps unthresholded for visualization). White arrow points towards the occipital pole; the arrow also indicates the direction of posterior to anterior. To test for cone-mediated retinotopic signals in visual cortex, rod-mediated polar angle estimates (*x*-axis) from left and right V1–3 are plotted against cone-mediated polar angle estimates (*y*-axis). If a cone map is present, data should cluster tightly around the identity line [dark blue dotted line; slope(β) = 1, intercept = 0], showing high correspondence and correlation across the two maps. Solid lines indicate the orthogonal linear regression fit to these data for ACHM patients pre-treatment (green), post-treatment (red) and normal-sighted controls (light blue). β = slope of fitted line; CC = Fisher–Lee circular correlation coefficient. Prior to treatment, patients had a well-defined rod-mediated retinotopic map, but the cone-mediated map lacks discernible structure. After treatment, a cone-mediated retinotopic map emerged for Patients Tr1 and Tr2, with rod/cone map similarity measures now resembling those of two representative age-matched normal-sighted controls [**C**(**i**) and **ii**)]. (**D**) Fisher–Lee circular correlation coefficient between rod and cone maps for 26 normal-sighted controls (blue) and 13 untreated patients with ACHM (green). Data for treated Patients Tr1–4 are also plotted individually: pre- (open green circles) and post- (open red circles) measurements of each patient are connected by a dotted red line. (**E**) Slope and intercept of rod–cone correspondence are plotted for the same groups as in **D**. Intersection of solid lines (β=1, intercept = 0) indicates high spatial correspondence between the rod and cone maps. Data from all normal-sighted controls (blue solid circles) cluster around this point, with the dotted ellipse indicating the 95% range of these data. Data from all 13 untreated ACHM patients (green solid and open circles) fall outside the normal range, as expected when cone signalling is absent ('Retinotopic organization of cone-mediated maps' in the Supplementary material). One untreated ACHM patient (Patient Utr8) had a very large negative slope and intercept and is therefore not plotted here (Table 1 and Supplementary Fig. 6A). Pre- and post-treatment measures for Patients Tr1–4 (green and red open circles, respectively) are connected by a dotted red line. For both correlation (**D**) and correspondence (**E**) results, indices of cone polar angle map structure fall within the normal-sighted range for Patients Tr1 and Tr2 but not for Patients Tr3 and Tr4.

During fMRI, participants viewed cone- and rod-selective stimuli embedded in a ring-and-wedge travelling checkerboard stimulus (Fig. 1C). We used a pRF modelling approach^36^ to measure cone- and rod-mediated retinotopic tuning. As expected, cone-selective pRF mapping evoked no visible polar angle or eccentricity map in untreated patients with ACHM, as visualized for Patients Tr1–Tr4 before treatment (Fig. 2A, bottom maps). In contrast and again as expected, the rod-mediated maps were clearly measurable in these same individuals (Fig. 2A, top maps). After treatment (Fig. 2B), a new retinotopic map mediated by cones had emerged in the visual cortices of Patients Tr1 and Tr2. These maps displayed upper (red) and lower (green) visual field representations in expected cortical locations, namely in similar locations as the upper and lower fields in the rod-mediated map. This provides striking evidence for new cone signalling in visual cortex of these two patients after gene therapy, albeit qualitatively. Patients Tr3 and Tr4, however, still showed no discernible cone-mediated polar angle map.

To move beyond inspection and statistically test for the existence of a retinotopic cone map, we took advantage of the fact that rod- and cone-mediated retinotopy is similar in spatial layout except around the foveal rod scotoma (i.e. the pRF estimates for each cortical location are similar regardless of whether we use a rod- or cone-stimulating stimulus).^37^ To test how similar cone-driven pRF position estimates were to rod-driven pRF position estimates, we examined the correspondence across the two polar angle maps. We did this by fitting an orthogonal linear regression model to rod versus cone polar angle map data, with high correspondence indicated by slope β=1 and intercept = 0 (identity line). We evaluated whether the maps displayed this predicted correspondence by testing whether the data were best predicted by the identity line (the correspondence model) or a horizontal or vertical line (each reflecting unstructured data in the cone or rod map) using the Akaike Information Criterion Weight (AIC_W_). The AIC_W_ represents the relative likelihood of the correspondence model versus the other two, with values close to 1 indicating strong evidence for correspondence and values near 0 indicating poor correspondence.^38,39^ We also examined the correlation across the two maps to test the reliability of the retinotopic map correspondence. We used the Fisher–Lee correlation coefficient (CC_FL_), as it accounts for the circularity of polar angle estimates.^40^

To generate a benchmark for the normal cortical signature of cone–rod correspondence, we used data from 26 normal-sighted children and adults, tested under identical circumstances. Figure 2C shows representative rod and cone polar angle maps, and rod-cone map correspondence and correlation for two normal-sighted control children age-matched to the treated patients. All controls showed strong evidence for correspondence (AIC_W_ ≈ 1), and a high correlation between rod and cone map polar angle estimates (CC_FL_ range = 0.28–0.89, *P* < 0.01 for all; see ‘Retinotopic organization of cone-mediated maps' in the Supplementary material for individual results). The individual measures for rod–cone map correspondence (β, intercept) and correlation (CC_FL_) for this group are shown in Fig. 2D and E, respectively, and normative ranges are indicated.

As expected, no measurable cone-mediated signals were present in visual cortex of Patients Tr1–4 before gene therapy: evidence for the rod–cone correspondence model was weak (all AIC_W_ ≈ 0) and correlations were low (Table 1 and Fig. 2). We also found no evidence for a cone-mediated polar angle map in the nine other untreated patients with ACHM (see Table 1 and ‘Retinotopic organization of cone-mediated maps' in the Supplementary material for individual results). Note that clustering of the cone-mediated polar angle estimates around zero in some of the untreated ACHM maps (Fig. 2A and Supplementary Fig. 6A) likely reflects a known fitting bias in the software unmasked by the absence of a retinotopic map, rather than true cone-mediated vision at this polar angle. Crucially, in all untreated patients including Patients Tr1–4, indices of spatial correspondence and correlation between the rod- and cone-mediated polar angle map fell well outside the normal-sighted range (Fig. 2D and E). This is in line with the lack of cone function in untreated patients with ACHM.

Promisingly, after gene therapy, correspondence and correlation measures from Patients Tr1 and Tr2 converged to show new evidence for an emerging cone-driven polar angle map in visual cortex: There was strong statistical evidence for the correspondence model (AIC_W_ ≈ 1 for both), and correlations between the rod and cone map data had improved into the normal range (CC_FL_ = 0.31, 0.60 respectively; Fig. 2B, D and E). It is important to note that it is exceedingly improbable for these indices of rod–cone map similarity to fall within the normal-sighted range without true presence of a cone map. This is because noise or bias in pRF estimation are unlikely to artefactually create the highly structured retinotopic pattern present in the rod-driven map (see simulations in Supplementary material). After treatment, Patients Tr3 and Tr4 still showed poor correspondence and correlation between their rod- and cone-driven maps (Table 1 and Fig. 2B, D and E). Importantly, changes in cone function after treatment in Patients Tr1 and Tr2 versus Patients Tr3 and Tr4 could not be explained by head- or eye-movement confounds: head- or eye-movement was not more stable after treatment than before in Patients Tr1 and Tr2, or compared to measures from Patients Tr3 and Tr4 (see ‘Eye movements’ and ‘Head movement’ in the Supplementary material). Furthermore, these pRF mapping results were replicated at a more stringent statistical threshold of *R*^2^ > 0.05 (‘Statistical thresholds’ in the Supplementary material).

Besides the polar angle, estimates of pRF tuning to positions in the visual field can be defined by the Cartesian *X*, *Y* coordinates and eccentricity. To test the robustness of the treatment effects we repeated the analyses for these other position estimates (‘Retinotopic organization of cone-mediated maps' in the Supplementary material). For Patient Tr2, rod–cone correspondence and correlation emerged for all these parameters and fell within the normal range after treatment. For Patient Tr1, rod–cone correspondence for all position estimates had improved post-treatment, but the values for the eccentricity and *Y* parameters did not reach the normal-sighted range ('Retinotopic organization of cone-mediated maps' in the Supplementary material). It remains plausible that cortical representations of certain parts of the visual field may have recovered to a lesser degree in this patient, as might be anticipated in this disorder which is characterized by variable potentially rescuable cone mosaics.

### Rod- and cone-mediated cortical visual field coverage

**Figure 3:**
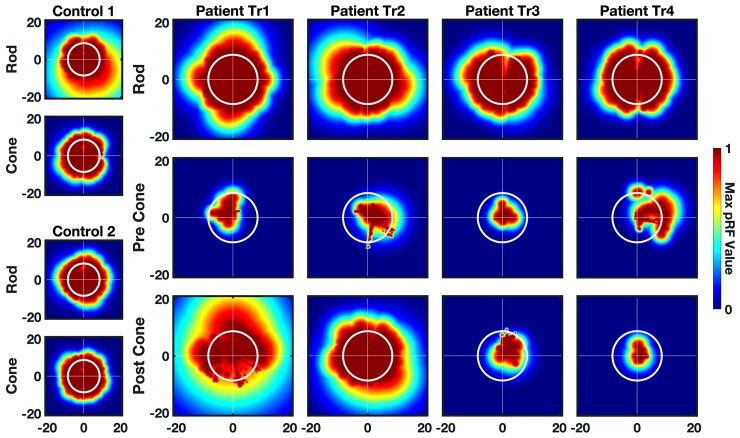
**Cortical visual field coverage.** Visual field coverage for V1–3 computed using the maximum pRF density for each participant across both hemispheres. *Left*: Visual field coverage of two representative normal-sighted controls (Controls 1 and 2) for rod and cone maps. *Right*: Coverage plots for rod map (pre-treatment) and cone map (pre- and post-treatment) for Patients Tr1–4. Colour indicates maximum value of pRF density. White circle indicates the maximum aperture of the stimuli (8.6°). Post-treatment cone-mediated visual field coverage encompasses the whole stimulated visual field for Patient Tr2 and is densest in the upper visual field for Patient Tr1.

To gain insight into the retinal locations driving the new cortical cone signals, we next evaluated cortical visual field coverage for the four treated patients (Patients Tr1–4, Fig. 3). Data from two representative normal-sighted controls (Ci and Cii) are included for comparison. Coverage was computed by plotting pRFs from V1–3 in visual field space and taking the maximum pRF density at each visual field location across both hemispheres for each participant and condition. Note that this approach is not optimal for mapping the foveal scotoma (Supplementary Fig. 2) as it does not show the ‘amount’ of field coverage in every location and even a single big foveal pRF in V3 would produce high values in big parts of the fovea.

In all participants, pRFs derived from rod-mediated retinotopic maps covered the full stimulated visual field, as did cone-mediated pRFs from the control participants. In contrast, cone-mediated visual field coverage in patients was poor before treatment. Note that the small areas of near-foveal coverage most likely arise from the pRF fitting bias mentioned above rather than central cone vision in untreated ACHM.

After treatment, visual field coverage had visibly increased in Patients Tr1 and Tr2, but not in Patients Tr3 and Tr4. In Patient Tr2 cone-mediated coverage after treatment was spread across the whole stimulated visual field and resembled cone coverage in normal sighted controls. In Patient Tr1, visual field coverage was strongest in the upper visual field, which may reflect more effective cone signalling from the lower retina.

### Rod- and cone-mediated population receptive field size

**Figure 4:**
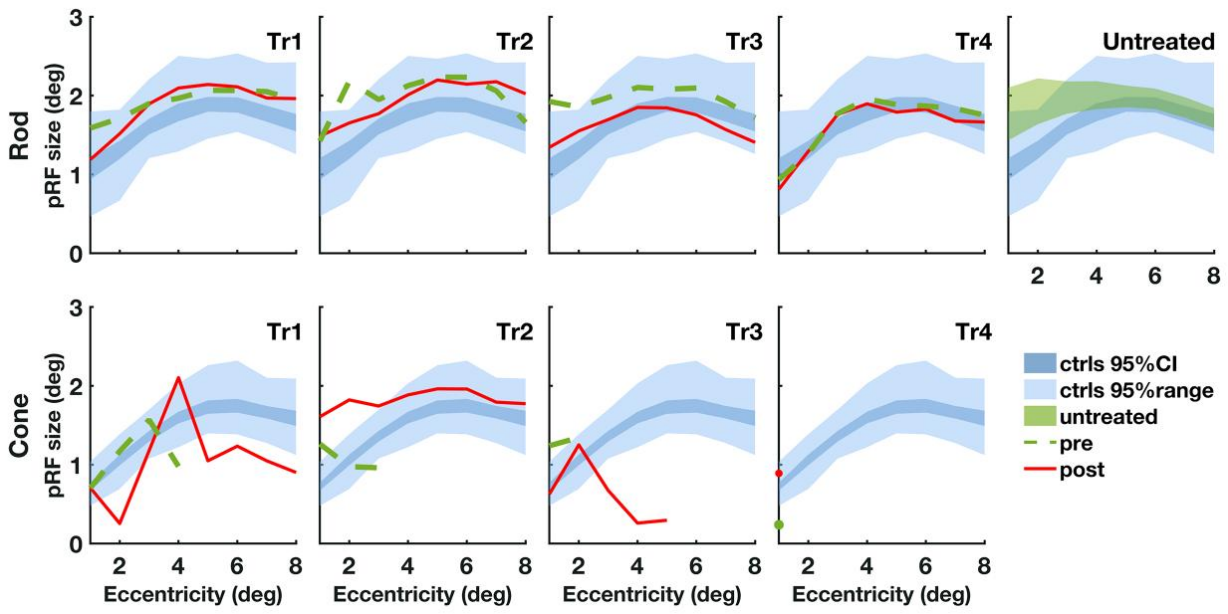
**pRF size by eccentricity.** Median pRF size for V1–3 across both hemispheres for each participant plotted against 1 degree eccentricity bins (range: 0.5–8.5). Bins with few data points were excluded (fewer than 10% of pRFs included in the equivalent bin in the non-selective condition). For each condition (rod, *top row*; cone, *bottom row*) the 95% CI (dark blue) and 95% range (light blue) of the normal-sighted control group (*n* = 26) are plotted. The 95% CI (green) of all 13 untreated patients with ACHM (*top right*) reveals ∼1 larger pRF size estimates compared to normal-sighted for small eccentricities. Pre-treatment data (dashed green line) and post-treatment data (solid red line) are plotted for treated Patients Tr1–4. There is no change in the rod-selective condition after treatment. In the cone-selective condition, pRF size can only be measured across the entire eccentricity range after treatment, which reveals small deviations compared to normal sighted for Patients Tr1 and Tr2 but no consistent increase or decrease across both patients. ecc = eccentricity; deg = degrees; pre = pre-treatment; post = post-treatment; ctrls = controls; CI = confidence interval.

We also investigated the relationship between pRF size and eccentricity for rod and cone maps in ACHM, and how it changes after gene therapy (Fig. 4). For each participant, and each condition, pRFs across V1–3 of both hemispheres were binned by eccentricities in 1-degree intervals (range: 0.5–8.5) and the median pRF size was calculated for each eccentricity bin. The range and mean (95% CI) of these measures for 26 normal-sighted controls (light and dark shaded blue, respectively) are shown for comparison. For the rod-selective condition, the 95% CI of the median pRF size across eccentricity bins is also plotted for all 13 untreated patients. In patient rod-mediated maps, we observed slightly larger average pRF sizes around small viewing eccentricities than in controls, differing by less than 1 degree. It is important to note that fixation instability has been shown to produce an overall increase of variability and also an increase in pRF sizes close to the fovea in amblyopia patients.^41^ This makes it hard to disentangle this confound from a true difference in pRF sizes between populations differing in gaze stability. Moreover, it is also possible that some differences arise from S-cone contributions in controls rod-mediated maps. We therefore conclude that rod-mediated pRFs in untreated ACHM are either similar in size to those of normal-sighted controls or slightly larger at small eccentricities.

Crucially, in the cone map, where the direct effect of treatment on cone-mediated function can be measured, the pRF size relationship was absent or atypical before treatment in all four patients with ACHM, in line with a lack of cone function. pRF size across all eccentricities could only be measured after treatment and in only two patients (Patients Tr1 and Tr2)—the same who showed evidence of new cone-mediated retinotopy, field coverage and perception. These new cone-mediated pRFs were slightly smaller (Patient Tr1) or larger (Patient Tr2) than in normal-sighted participants. This may in part reflect higher measurement variability in ACHM and for new cone-mediated pRFs after gene therapy. Together these data suggest that pRF size measures of new cone-mediated cortical signals may be more variable in treated patients compared to individuals who had cone vision from birth.

## Discussion

We provide the first demonstration that retinal gene therapies can recover cone photoreceptor signalling in the neural pathways of children born with a monogenic disease that disrupts cone function from gestation (ACHM). To assess this, we developed a novel approach that combines fMRI and psychophysics with stimuli that allow us to separate changes in the treated cone photoreceptor system from existing rod function. We tested four children (aged 10–15 years) with ACHM undergoing gene therapy and compared their results with measures from nine additional untreated patients and 28 normal-sighted controls. We found converging evidence for recovered cone function in two of four treated patients, showing that it is possible for a gene therapy to engage these dormant visual streams in the human brain despite many years of deprivation.

We first established that our measures correctly isolated cone-mediated information processing channels from rod-mediated channels. As expected, in behavioural tests of cone function, all untreated patients (the four undergoing gene therapy and nine in a larger untreated group) performed at chance level when detecting stimuli with chromatic contrasts that selectively activated cone photoreceptors and were indistinguishable for rods (Fig. 1). In contrast, all normal-sighted controls (*n* = 28) detected these cone-selective stimuli effortlessly. Untreated patients with ACHM also demonstrated no measurable retinotopic cortical responses during cone selective pRF mapping (Fig. 2D and E). This was despite the rod-selective chromatic contrast evoking a clear polar angle map in each patient. Thus, untreated patients with ACHM displayed no evidence of cone function across psychophysical and neural measures.

Six to 14 months after gene therapy, the cone-photoreceptor targeting stimuli had become discriminable to two of the four treated patients (Patients Tr1 and Tr2). Selective to the treated eye, Patients Tr1 and Tr2 had improved to ceiling level on two psychophysical tests of cone contrast sensitivity. Strikingly, there was strong evidence for cone-driven signals in visual cortical areas V1–3 of both patients, which had been absent prior to treatment. These new cone-mediated activation patterns also showed the clear retinotopic organization that is a hallmark of functional vision in normal-sighted individuals, with high correspondence and correlation between the rod and cone maps. Indices of retinotopic tuning to cone signals in visual cortex of Patient Tr2 fell within the normal range after treatment. While cone-mediated position tuning had clearly emerged in the visual cortex for Patient Tr1, measures fell outside the normal range, perhaps reflecting better cone function in selected visual field locations ('Statistical thresholds' in the Supplementary material). The two other treated patients (Patients Tr3 and Tr4) showed no change on these psychophysical and fMRI measures of cone function, potentially reflecting individual differences in treatment effects. Together these findings provide converging evidence from psychophysics and functional fMRI for new cone-mediated signalling between the retina and visual cortex after gene therapy.

We next explored whether retinocortical cone signalling after gene therapy was fully or only partially recovered across the visual field. Analyses of cortical visual field coverage revealed that in Patients Tr1–4, rod-mediated pRFs in V1–3 tiled the entire stimulated area as in normal-sighted controls, while cone-mediated pRF coverage before treatment was highly restricted. After treatment, cone-mediated visual field coverage in Patients Tr1 and Tr2 was in keeping with the emergence of cone-mediated retinotopic maps in these same patients. In Patient Tr2, the cone-mediated pRFs covered the full visual field following intervention. In Patient Tr1, coverage was most dense in the upper visual field, potentially reflecting more effective gene therapy targeting of the lower retina, and/or variable therapeutic potential of the retina in different locations based on variable cone integrity.

Our cone- and rod-selective stimuli also allowed us to test, for the first time, how pRF size (often taken as a proxy for cortical spatial acuity) is affected in ACHM before and after gene therapy. Rod-mediated pRF sizes in the visual cortex of untreated patients resembled those of normal-sighted controls, with slightly larger pRF sizes at near-foveal eccentricities (<1 degree difference). This enlargement may reflect atypical pRFs around the normal rod scotoma, but may also be an artefact of nystagmus or an unintended cone response to our rod-selective stimulus in controls (see ‘Validating rod selective stimuli’ in the Supplementary material). For cone-selective stimuli, we could only record a pRF size/eccentricity relationship after treatment in Patients Tr1 and Tr2—there were insufficient data in all other patient measures. The new cone-mediated pRFs deviated slightly in size from those measured in normal-sighted in both patients, but differences were small (<0.5 degree) and inconsistent and may in part reflect greater measurement variability for new, weaker cone-mediated pRFs in ACHM. Thus, rod- and cone-mediated pRF sizes in ACHM did not differ by large degrees from those in the normally developing visual cortex, with the observed small differences needing to be confirmed with more data and controls for nystagmus.

Given the highly organized nature of retinotopic responses in early visual cortex, it is most unlikely that the treatment-related changes in cone-mediated spatial tuning that we report could have emerged from random noise fluctuations (as shown via simulation in ‘Statistical thresholds’ in the Supplementary material). In addition, head and eye movements were measured throughout the pre- and post-treatment scan sessions and were relatively low and comparable across conditions and time points (‘Eye movements’ and ‘Head movement’ in the Supplementary material). Moreover, given the concurrent change in psychophysics measures of cone function, which unlike fMRI did not require fixation or lying still, the individual differences in cone-mediated function in Patients Tr1–4 are unlikely to be explained by nystagmus or head movement. It cannot be determined from current data why Patients Tr1 and Tr2 had stronger treatment benefits on our tests than Patients Tr3 amd Tr4. Patients Tr1 and Tr2 were not younger, had no better visual acuity and had not had more time to recover from treatment (Table 1) than Patients Tr3 and Tr4. Differences in cone function recovery after therapy may depend on other pre-existing genetic-, retinal-, post-retinal- or treatment-related predictors to be explored further in future studies.

From these early but striking results, we can conclude that gene therapy can successfully activate the dormant cone photoreceptor pathways in ACHM after 10–15 years of life, and evoke visual signals not previously experienced by these patients. Moreover, our data promisingly suggest that retinotopic spatial tuning to cone-mediated signals can be achieved in visual cortex after gene therapy. This work provides the first direct demonstration that, at least in some cases, a degree of neural infrastructure for useful cone function is preserved in ACHM after extended deprivation, well beyond most sensitive periods for vision. These results are in line with other studies on visual deprivation. For example, the removal of congenital cataracts is most effective when done early in life, but some visual recovery is still possible even if treatment occurs late or in adulthood.^42,43^ In the case of ACHM, experience with rod-based vision may scaffold perceptual capacities that late-recovered cone-mediated function may benefit from.

Our findings differ notably from those of a recent study that measured pRFs in two adults with *CNGA3*-linked ACHM undergoing gene therapy. Before treatment, these patients displayed highly atypical rod-mediated eccentricity maps with parietally shifted foveal confluences, and markedly enlarged (>4 degrees) pRF sizes in cortex normally containing V1–3. After treatment, the pRF sizes were reduced. While McKyton *et al.*^10^ used standard pRF mapping stimuli that activate rods and cones non-selectively, they speculated that the pRF size reductions were due to treatment-induced cone function. However, unlike this prior study, we observed no reductions in pRF size after treatment for stimuli that activated rods and cones simultaneously (reported in ‘pRF sizes with non-selective stimuli’ in the Supplementary material), even though Patients Tr1 and Tr2 showed strong evidence for new cone function after gene therapy on other indices. Our results also differed from those of McKyton *et al*.^10^ in that we found more typical rod-mediated retinotopic maps in untreated ACHM, with the foveal confluence of the eccentricity map in the expected location near the occipital pole. Like McKyton *et al*.,^10^ we found enlarged pRF sizes in untreated patients with ACHM compared to controls for stimuli that activated rods alone as well as for stimuli that activated rods and cones (Supplementary material). However, in our data the enlargement was drastically smaller (1 degree difference) and only present at small viewing eccentricities, unlike the previously reported ∼4 degrees difference across all eccentricities. These differences across the studies are unlikely to be due to patient age, as rod-mediated pRFs had similar sizes in the children and adults in our untreated patient sample (Table 1 and Supplementary material). They may reflect different light levels used during scanning. While we stimulated photoreceptors at low luminance (maximum 0.5 cd/m^2^) to ensure rods and cones were both sensitive and viewing was comfortable for light-averse children, McKyton *et al*.^10^ used bright stimuli (maximum 180 cd/m^2^) that likely saturated rods. They also did not record gaze during scanning and as mentioned before fixation instability has been shown to increase pRF size variability and size.^41^ Either factor may have contributed to why we did not replicate the treatment-related changes in pRF size.

After demonstrating that cone signalling in the visual cortex can be successfully restored and used to improve cone contrast perception, an important next question is how this information can be used for the more complex visual functions that the cone system normally feeds into. Incidentally, Patient Tr2 reported seeing ‘better’ with their treated eye, mentioning perceived benefits for reading traffic signs. However, visual acuity specific to the treated eye did not significantly improve in treated patients, suggesting patient-reported benefits were not well-captured by this measure. In future studies, comparison with other clinical assessments, neuroimaging measures and subjective benefits will be crucial for understanding how recovering cone function can benefit patient vision in the broadest sense.

An important related question is how effects of gene therapy can be maximized. While new cone function had clearly emerged in two treated children, their measures remained less robust than those of normal-sighted controls: in both patients, discrimination was slower although accuracy was at ceiling, cone-mediated signals in the visual cortex were weaker (pRF goodness of fit was lower) and for Patient Tr1, cone-mediated signals appeared non-homogeneous across the visual field. Normal levels of cone function are unlikely after gene therapy given the long period of deprivation in even these younger patients and the markedly reduced cone densities and variable topography characteristic of ACHM, but effects may also be limited by ocular competition: by trial protocol, only the worse eye with lowest acuity was treated, while our data were collected binocularly. Our measures were therefore susceptible to potential competing vision from the untreated better and typically dominant eye, and monocular measures may record larger changes in cone function. It is also possible that treatment benefits themselves are reduced by competition for neural resources from the untreated eye or rod system. Neuroplasticity after therapy may be enhanced by counteracting such effects, via post-treatment patching of the untreated eye, or training with cone-selective stimuli, or binocular therapy. We anticipate that the child-friendly measures of cone function reported here will be critical for elucidating how genetic, neural and developmental factors interact with regenerative therapies to support visual recovery in patients with ACHM, and the many other congenital eye diseases for which new treatments are currently under development.

In conclusion, our work is the first to directly demonstrate that gene therapy during visual development can successfully activate the dormant cone photoreceptor pathways and evoke visual signals never experienced by children with ACHM. This holds great promise for worldwide efforts to translate gene replacement technology to human patients.

## Supplementary Material

awac226_Supplementary_Data

## Abbreviations

ACHM: achromatopsia
pRF: population receptive field
