# Supplementary material for: A demonstration of cone function plasticity after gene therapy in achromatopsia
Source: Brain. 2022 Aug 24;145(11):3803–15. doi: 10.1093/brain/awac226 (PMC9679164; doi:10.1093/brain/awac226)
Supplement: awac226_Supplementary_Data [file awac226_supplementary_data.pdf]

## Supplementary Materials 1: Validating cone selective stimuli

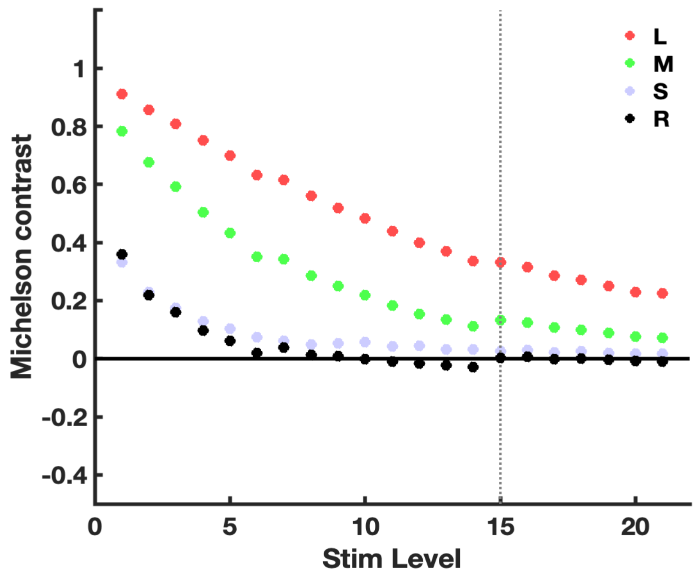


*Figure S1a. Cone selective stimuli: Michelson contrast for L-, M-, S-cones and rods, across chromatic pairs designed to selectively activate L and M cones. Contrasts are shown for 21 stimulus intensities designed to gradually decrease L + M contrast. Black solid line indicates zero contrast. Black dotted line indicates the contrast of the stimulus presented in the scanner.*

Cone selective chromatic pairs (shown in Main Figure 1A, B) were designed to increase (foreground) and decrease (background) cone contrast in 20 steps (see Main Methods). To quantify photoreceptor-specific contrast induced by each pair, we measured the screen spectral output for each RGB triplet used to increment/decrement the cone response, using a Spectrascan Spectroradiometer (PR-655, PhotoResearch Inc.). Measures were corrected for distortion from neutral density filters and the MRI mirror. Each of the chromatic spectra were then multiplied with the standard observer photoreceptor fundamentals for long (L)-, mid (M)-, short (S) cone and rod (R) photoreceptors (Stockman & Sharpe, 2000; Wyszecki & Stiles, 1982) resulting in 2 activation levels (background vs. foreground) per chromatic pair for each photoreceptor type. To quantify photoreceptor-specific contrast between the foreground and background of each chromatic pair, we computed the Michelson contrast, with values near zero indicating minimal photoreceptor activation (*i.e.,* ‘silence’). Figure S2a displays the L, M, S, and R Michelson contrast across 21 chromatic pairs, designed to gradually decrease L and M cone contrast, whilst keeping rod contrast zero. As intended, the computed contrast for L-cones and M-cones decreased gradually across the stimulus range. S-cone contrast (left uncontrolled) was substantially lower, and also decreased across chromatic pairs. Figure S1a also reveals imperfect silencing of rod photoreceptors for higher stimulus contrast levels, likely due to technical issues related to imperfect correction for the non-canonical gamma function applied by the MR-compatible LCD display. Therefore, patients without

functioning cones were a-priori expected to perceive the higher-contrast stimulus levels using rod-based vision. In addition, this approach rests on the assumptions that photoreceptor sensitivity functions are identical across ACHM patients, normal sighted controls, and retinal eccentricities, and that light measurements are precise and accurate. In reality, variations in photoreceptor sensitivity and measurement error, may induce deviations from the photoreceptor activation levels shown in Figure S1a, and thus limit our ability to control and verify individual photoreceptor activation levels.

Given these considerations, it is crucial that we were able to establish that chromatic pairs with lower L + M contrasts, including the contrast shown in the fMRI scanner (Level 15, shown as the vertical dotted line in Figure S1a and as the horizontal dotted red line in Figure 1C&D), were below the detection threshold for all the untreated patients with ACHM, across the two psychophysical tasks. These individual threshold estimates had good correspondence across the two tasks (Figure S2b), even though the chromaticity pairs were embedded in spatiotemporally different stimuli and different tasks (locating a target versus discriminating checkerboard movement, Main Figure 1). Data collected in piloting and a subset of study patients, showed that measures were also repeatable across staircase starting points (i.e., starting at the dimmest or the brightest contrast level). Note that for normal sighted individuals, all contrasts included in the cone-selective stimulus range were above threshold and clearly visible, with performance at ceiling (level indicated by yellow square Figure S1b).

Together these measures show that the range of cone-selective stimuli used in our psychophysical tests and pRF mapping were correctly calibrated to stimulate L and M cones but not rods. We therefore conclude that improvements in contrast sensitivity beyond the pre-treatment range of performance, provides strong evidence for emerging cone function in treated ACHM.

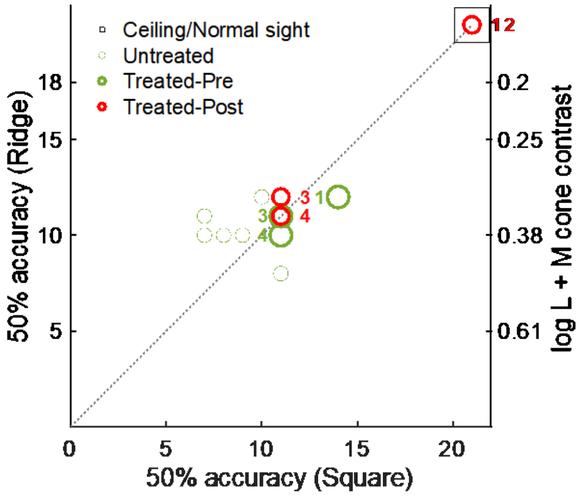


*Figure S1b: Two 50% accuracy cone sensitivity thresholds were measured for 10 untreated patients with ACHM using the range of L&M contrasts shown in Figure S1a. These thresholds were obtained using two tasks: the 4AFC square localisation psychophysics task that involved locating a target square (Square; see Figure 1A) and a Ridge motion discrimination task that involved judging movement direction of a ring-and-wedge stimulus (Ridge; see Figure 1B). Thresholds on each measure for untreated patients in Main Figure 1 (n=10) are scattered against each-other in green, to visualise the test-retest range across both tasks. Notably, there was good correspondence across threshold estimates despite the different tasks and chance-performance levels for the Square and Ridge(mean threshold difference = 0.45, maximum difference, 4 steps). Patient Tr1, Tr3, and Tr4's pre-treatmetn measures are indicated by green unfilled circles (we did not collect a pre-treatment measure for Tr2). Tr1 and Tr2's post-intervention measurement (red unfilled circles) fell well outside this two-dimensional range, and was at the ceiling level reached by normal sighted controls.*

## Supplementary Materials 2: Validating rod selective stimuli

To match the rod-mediated retinotopic map as closely as possible across individuals with and without cone function, we generated RGB triplets that increased and decreased rod activation, whilst keeping L and M cone activation minimal (See Figure S2A; Estévez & Spekreijse, 1982; Spitschan & Woelders, 2018). When using three colour channels (R, G, B) only two photoreceptors types (here two cone types) can be silenced simultaneously, so there was substantial S-cone contrast for the rod-activating chromatic pairs. In addition, there was some unintended L and M cone contrast, likely due to light measurement- and gamma correction error. To mitigate these unintended cone signals in the scanner, we presented stimuli at the darkest light level possible (0.02 cd/m$^2$, low mesopic) to favour rod responses and render cones insensitive. The rod-mediated eccentricity map obtained with these stimuli, averaged across 26 normal-sighted control participants (all cortical surface-based aligned to fsaverage), revealed signal loss around the fovea (Figure S2B, left) compared to the cone-selective map in these same individuals. This suggests that we successfully mapped the central rod scotoma, by minimising, if not eliminating, responses from cone photoreceptors, which primarily populate the fovea (Figure S2B, right).

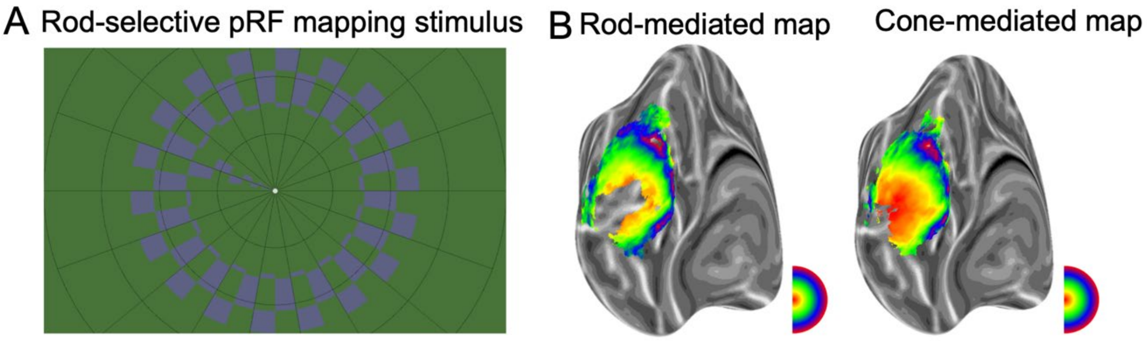


*Figure S2. A) Rod-selective pRF mapping stimulus shown in the MRI scanner. Participants detected target dimming events at fixation. B) Left: rod-mediated pRF map, obtained with stimulus in (A) averaged across 26 normally sighted control participants. Right: Cone-mediated pRF map obtained with stimulus in Main Figure 1C, averaged across 28 normally sighted control participants. Loss of signal around the fovea in the rod-mediated map compared to the cone-mediated map (areas coded red), suggests cone responses around the fovea were successfully reduced by the rod-selective stimulus. Both maps are generated by co-registering every control participant's individual cortical surface to a common cortical surface (the fsaverage) using cortex-based alignment as implemented in Freesurfer. Functional pRF mapping data was sampled to this common cortical space, thresholded at $R^2$>0.05, and averaged across participants. In areas not colour-coded by eccentricity, none of the control participants had a pRF fit that survived the statistical threshold.*

## Supplementary Materials 3: Eye movements

Eye tracking with the Eyelink 1000 relies on a built-in calibration routine, which requires high levels of fixation stability. Patients with ACHM, especially when younger, experience involuntary eye movements of varying amplitudes due to nystagmus. This makes it challenging to complete this built-in calibration. To map gaze coordinates of patients in this study to screen locations during the fMRI scans, we conducted a post-hoc calibration procedure as used recently with patients with congenital nystagmus (Tailor et al., 2020), similar to other procedures (Dunn et al., 2019; Rosengren et al., 2020). We first ran a pre-calibration of the eye-tracker in the scanner using the built-in calibration on a normal sighted individual. When placing patients with ACHM in the scanner, their eye was placed in a similar position as the 'pre-calibrated' eye. To account for individual differences in eye characteristics and positioning, we ran an additional in-house calibration with patients; On every other run during scanning, we asked the patients to fixate on a target presented in 5 positions across the screen in a random order (at fixation and above, below, left, and right of fixaton at 5° eccentricity). The participant was instructed to look at the fixation target for 5 seconds and move to the new location when it appeared. We then used these recordings to perform a post-hoc calibration of the data.

**Calibration:** In post-hoc calibration, we first applied a stringent procedure to remove blinks and eye movements from the 5-point calibration recordings and identify the fixation locations for each calibration point. We first removed the first 0.5 seconds for each fixation location to allow for fixation to arrive on the target. We then removed (a) blinks and the 0.15-second period before and afterwards, when the eyelid closes and opens, (b) eye movement velocities that fell 2SD above or below the mean velocity, and (c) any positions >3SDs to the left or right of the mean fixation location, and >1SD above or below. We took the median of the remaining gaze measurements as an approximate fixation estimate. The resulting 5 median fixation locations were used to fit an affine transformation that remapped the recorded gaze positions into screen space.

**Quantifying fixation stability:** To assess fixation stability, we applied the affine transformation obtained from the post-hoc calibration to the gaze positions recorded during the central fixation task for pRF mapping. Using data that was unfiltered for blinks and other eye movements, we first verified good compliance with the fixation task (Figure S3a). Because vertical gaze position during scanning is confounded with blink-, eye-lash- artefacts, and drift from scanner vibration, we focussed on the horizontal (x) direction, which is the dominant direction of nystagmus. Fixation compliance was high, in line with high fixation task performance (>95%), and good fixation

observed on a face camera. Then, to quantify eye-movement amplitude in the horizontal direction, we removed blinks and eye movement velocity outliers (±2SD). After applying this filter, characteristic pendular nystagmus was discernible in ACHM patients (see Figure S3b, top panel for example). To obtain an index of fixation stability per condition and session for each participant, we computed the standard deviation of gaze position across consecutive 1-second sections of these horizontal eye position measures (see Figure S3b, bottom panel for example), and then took the median of these measures across run 1 and 2. Results for the 4 treated are reported in Table S3. Results for all controls, untreated patients with ACHM and Tr1-4 are plotted in Figure S3c. In our data for all groups, fixation stability was comparable across conditions, although patients' fixation was less stable of that of controls. Fixation stability for Tr1-4 measured throughout the pre- and post-treatment scan sessions was relatively low and comparable across conditions and time points. In addition, we can use the rod-selective condition in the untreated group as a benchmark to a level of acceptable fixation stability as for these participants, in this condition a retinotopic map has been obtained. Post-treatment fixation measures in the cone-selective condition of Tr1-4 fall well within that range. Based on these measures we conclude that the differences in cone-mediated map structure in the cortex of patient Tr1-4 before and after treatment are highly unlikely to be driven by different fixation stabilities. Moreover, given the concurrent change in psychophysics, which unlike fMRI does not require good fixation, the longitudinal differences in cone-mediated function in patients Tr1-4, are unlikely to be explained by nystagmus.

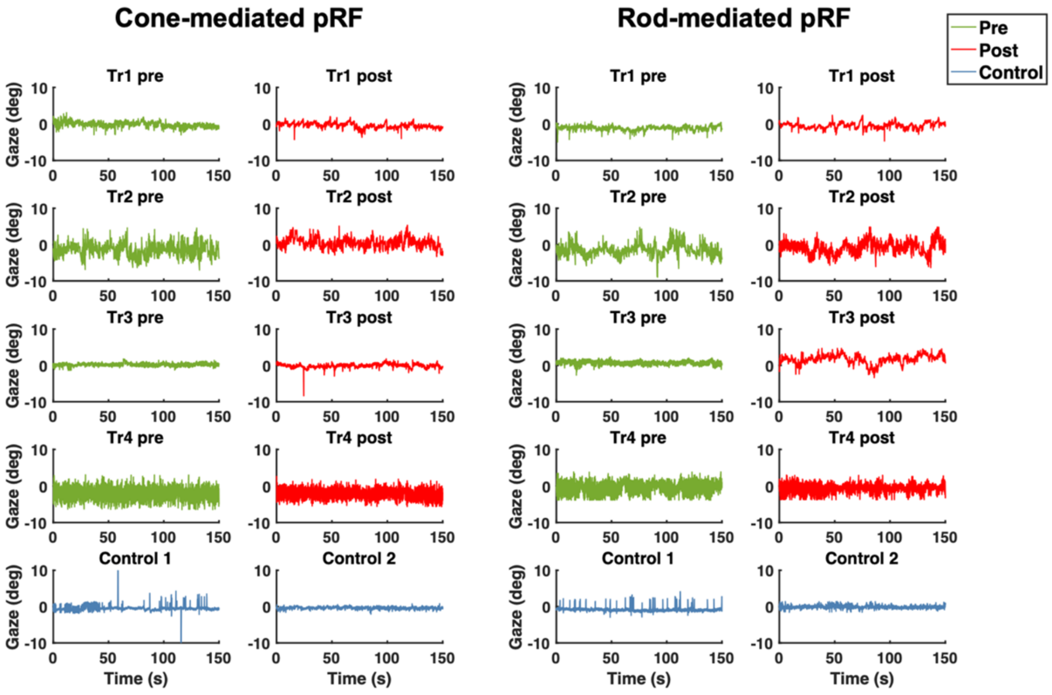


*Figure S3a. Unfiltered horizontal gaze measures across the first 150 seconds of scanning (out of 340 seconds) during the 1st data acquisition run for the cone map (1st out of 2) in patients Tr1, Tr2, Tr3, and Tr4 compared to the 2 control participants.*

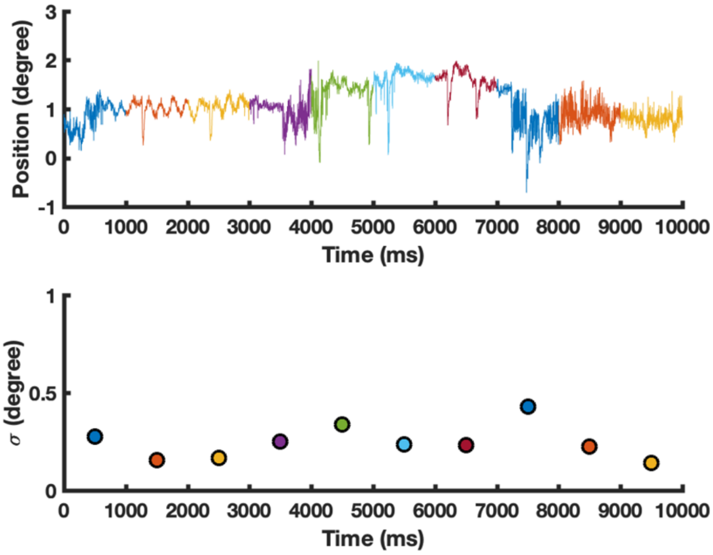


*Figure S3b. Top: Horizontal eye movement recordings from patient Tr1 during fMRI scanning, after the removal of blinks and recordings with velocity exceeding 2SD. Data are from first 10 seconds of fixation during the first rod-selective pRF mapping scan. 1-second consecutive sections used to compute standard deviation of gaze position in panel below are colour-coded. While a characteristic pendular movement is visible, the amplitude is very small, in line with that this participant had good control over their nystagmus. Below: standard deviation computed per 1-second interval of data, across colour-coded 1-second intervals in top panel.*

| | Tr1, pre | Tr1, post | Tr2, pre | Tr2, post | Tr3, pre | Tr3, post | Tr4, pre | Tr4, post |
|---|---|---|---|---|---|---|---|---|
| **Rod Stim** | 0.19 | 0.25 | 0.85 | 0.75 | 0.17 | 0.27 | 0.93 | 0.69 |
| **Cone Stim** | 0.24 | 0.22 | 0.84 | 0.63 | 0.18 | 0.15 | 1.25 | 1.00 |

**Table S3: Median standard deviations of horizontal eye movement across 1-second sections along the two 340-second runs, after blink- and velocity outlier removal (±2SD).**

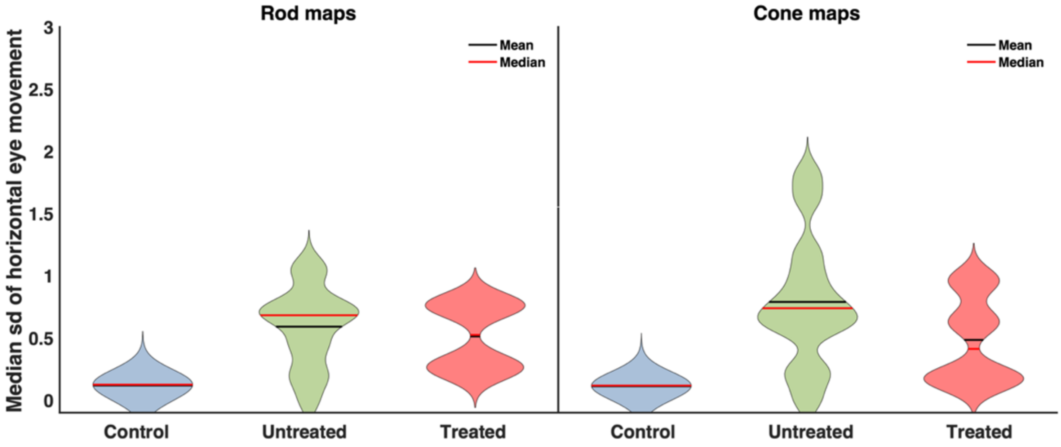


*Figure S3c. Group level fixation stability comparison. For each participant, condition and run, the median standard deviations of horizontal eye movement across 1-second sections were calculated. Data for both runs of each participant is plotted for the Rod map (left) and Cone map (right).*

## Supplementary Materials 4: Statistical thresholds

The key fMRI outcome measure in this report is the emergence of a cone map in patients with ACHM. We tested this by taking advantage of the fact that rod and cone-mediated retinotopic maps are highly similar in spatial layout (i.e., the pRF parameters of each cortical location are similar for rod- and cone-mediated mapping stimuli). This can be shown qualitatively by inspecting the maps themselves. Here we move beyond subjective inspection and quantify the existence of cone maps quantitively by testing for high correspondence (using linear regression) and the reliability of this correspon(using correlation tests) across the two maps. It is important to note that use of a low statistical threshold for this analysis does not bias the result if this simply adds more noise, because unstructured noise is unlikely to give rise to artefactual appearance of a retinotopic map.

This can be illustrated with a Monte Carlo simulation that computes the chance of finding high correspondence and correlation between pRF estimates of the rod and cone map in the absence of a true underlying cone map. We simulate the absence of a cone map in two ways: (1) by randomly shuffling the values of a normal sighted control cone map, and (2) by doing the same for the cone map of an untreated patient with ACHM, both without any statistical threshold applied ($R^2>0$). Not a single iteration of these simulations resulted in a linear correspondence (parameterised by an intercept, and slope) or correlation (Fisher-Lee correlation coefficient) that fell anywhere within the range observed in normal sighted control participants. Thus, the chance of detecting a cone map if no such map present in visual cortex, simply due to random fluctuations in the measure, is smaller than 1 in 10,000 (the number of iterations run). This shows that a low statistical threshold for these analyses is unlikely to compromise the accuracy of the results. Rather, applying a low or no statistical threshold may increase the chance of detecting potentially weak cone-driven retinotopic patterns in fMRI data. In line with these simulations, our results remained similar when testing for cone maps at lower or higher thresholds than $R^2>0.03$, reported in the main text ($R^2>0$ and $R^2>0.05$).

Figure S4a shows that the results of the analyses for cone-mediated retinotopic signalling in visual cortex presented in Main Figure 2 remain similar when using only data for which $R^2>0.05$. Figure S4b shows that the results of the visual field coverage analyses presented in Main Figure 3 remain similar when applying a statistical threshold of $R^2>0.05$. Figure S4c shows that the same applies to the pRF size analysis, with one exception: when binning, averaging, and plotting pRF size against eccentricity for Main Figure 3, we excluded eccentricity bins in which fewer than 10%

of the pRFs counted in the rod map survived the statistical threshold. This was to avoid unreliable estimates of median pRF size based on small numbers of datapoints in a bin. At $R^2>0.05$, many eccentricity bins for Tr1 do not meet these additional exclusion criteria. This shows that whilst significant new cone-mediated retinotopic signalling was present for this patient after the gene therapy (as confirmed by converging evidence from retinotopic maps and behavioural measures), this cone-mediated signal had relatively low $R^2$ compared to cone-mediated signals measured for Tr2 after gene therapy, and to normal sighted controls.

In sum, these analyses show that our key finding of the emergence of cone-mediated cortical signalling after gene therapy in 2 out of 4 treated children with ACHM, cannot be explained as an artefact of statistical threshold selection.

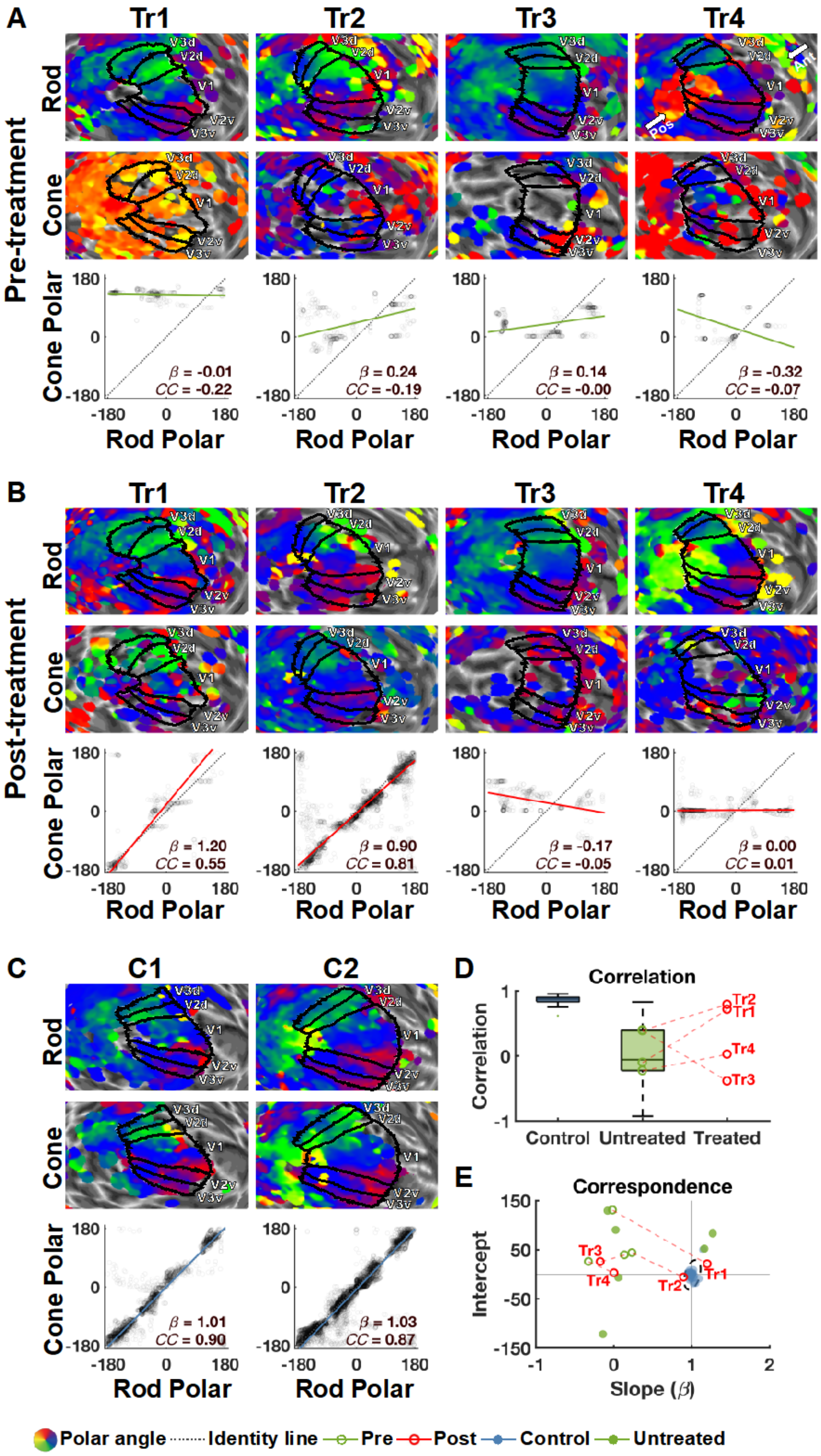


*Figure S4a: Same as Main Figure 2, but only including pRF fits with $R^2$>0.05 instead of $R^2$>0.03. Note that the patterns of results remain similar with this more stringent statistical threshold.*

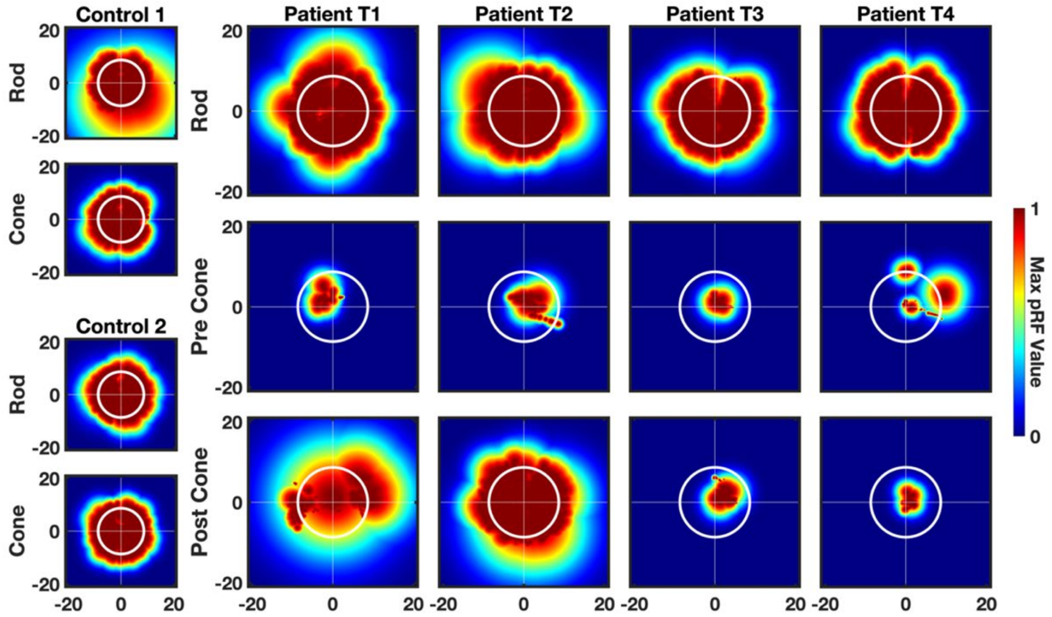


*Figure S4b: Same as Main Figure 3, but only including pRF fits with $R^2$>0.05 instead of $R^2$>0.03. Note that patterns of results remain similar with this more stringent statistical threshold for all participants.*

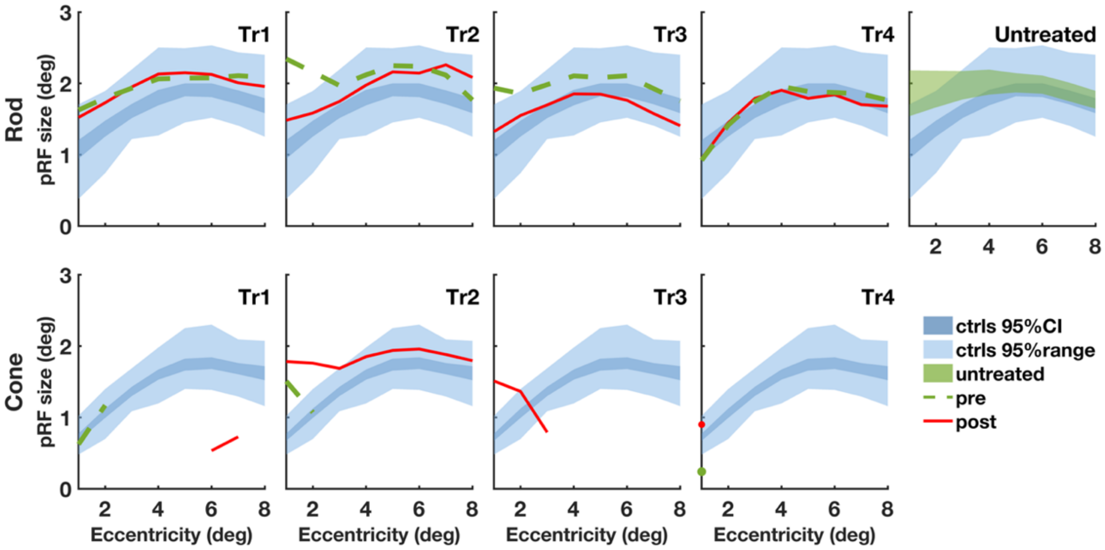


*Figure S4c: Same as Main Figure 4, but only including pRF fits with $R^2$>0.05 instead of $R^2$>0.03. Note that the patterns of results remain similar with this more stringent statistical threshold. Note also that we employed the criterion that for the cone-mediated pRF data from an eccentricity bin to be included in the plot, 10% of datapoints measured in that same eccentricity bin with the non-selective stimulus must be retained. At this higher threshold, much of the cone-mediated data, including that for Tr1 after treatment, no longer survive this stringent requirement.*

## Supplementary Materials 5: Head movement

Head motion can affect the quality of fMRI data. It is therefore important to make sure that there were no significant differences in head motion between the groups, between patients who showed an effect and those who did not, and between pre and post scans within a patient.

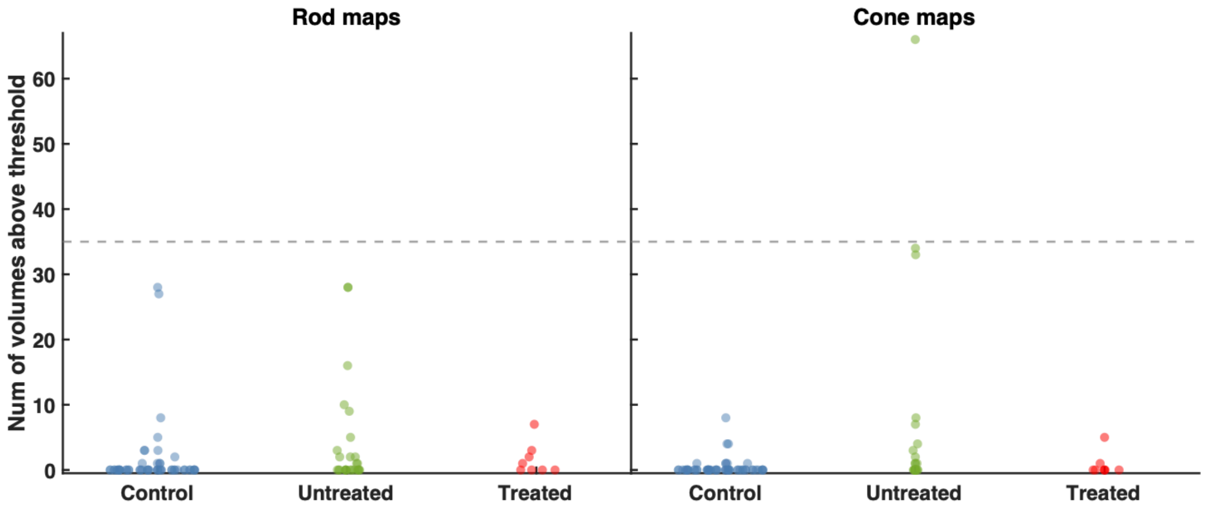


*Figure S5a. Group level head motion comparison for controls, untreated patients with ACHM, and patients Tr1-Tr4. Frame displacement is the total amount of movement the head made from volume to volume. For each participant, condition and run, the number of frame displacements higher than 0.9mm was calculated (Note that these are still sub-voxel movements as the voxel size was 2.3mm isotropic). Dotted line denotes the threshold of an acceptable quality run (10% of volumes = 35 volumes). Each dot represents a single run, therefore, in each plot (Rod: left, Cone: right) includes 2 dots for each participant representing run1-2 of that condition.*

First, any small head movements and slow drifts, such as gradual downward drift, were accounted for using standard motion correction procedures as implemented in SPM12. Then, a 'usable' run was defined as a run that has less than 10% of volumes with less than 0.9mm frame displacement (dotted line in Figure S5a; Siegel et al., 2014). Head motion is a known challenge when scanning children with fMRI paradigms. In our data for all groups, in conditions where a retinotopic map was expected (Rod and Cone in controls, and Rod only in untreated patients with ACHM), data with movement below this threshold produced a clear measurable map (see Figure 2 & Supplement 4). This demonstrates the robustness of our approach even in paediatric participants. One untreated baseline patient had one run (in their cone-selective condition) with movement above the threshold, however none of our reported results change when excluding this participant. Crucially, all treated participants had very good head stability with less than 7 volumes (~2%) of movement above the threshold in either pre or post runs (See Figure S5b). The patients that showed evidence for improved cone function after treatment (Tr1&2) did not produce less head motion than those who did not show an effect (Tr3&4). Moreover, our key finding, the emergence of a highly structured retinotopic map after gene therapy, is highly unlikely to originate from head motion

artefacts, as head movement was low in each of these patients and well-matched across pre- and post-treatment measures in Tr1-4.

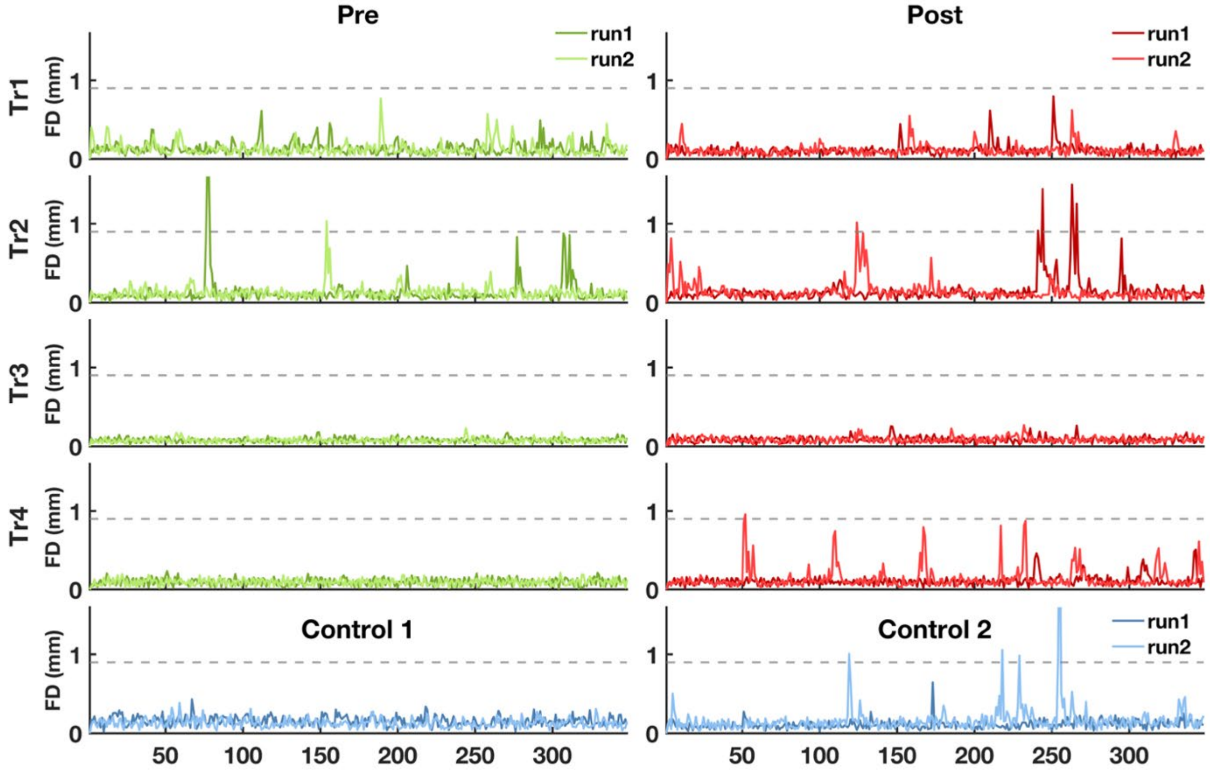


*Figure S5b. Online head motion for pre- and post-treatment fMRI data. Frame displacement (FD) over time is plotted for each condition and session for Tr1-4 and two representative controls. Dotted line denotes the threshold criteria 0.9mm.*

## Supplementary Materials 6: Retinotopic organisation of cone-mediated maps

We estimated cortical visuospatial tuning to cone- and rod-selective stimuli using a pRF mapping approach, to investigate cone-mediated retinotopic signalling in visual cortex after gene therapy. We tested for the presence of retinotopic structure in the cone map by comparing cone-mediated pRF position estimates against rod-mediated pRF position estimates. Specifically, we used linear regression to test for high *correspondence* (whether rod and cone map position estimates scattered against each other follow a line with slope of 1 and intercept of zero) and correlation analyses to test for high *correlation* (a reliable correspondence indicated by tight clustering of data along the fit regression line) across the two maps.

In Main Figures 2A-C, we report these analyses for 4 paediatric patients Tr1-4 before and after they underwent a gene therapy aimed at inducing retinal cone function, and for 2 age-matched control children. In Figure 2D&E, we report correlation (Fisher-Lee correlation coefficient) and correspondence (regression slope and intercept) indices of the 24 normal-sighted controls and 9 untreated patients with ACHM. In Figure S6a, we plot individual scatter plots for all normal sighted controls as well as untreated patients with ACHM. For all normal sighted controls, the data was best explained by a correspondence model ($AIC_w \approx 1$), in line with presence of a cone-mediated retinotopic map. In contrast, for all untreated AHCM patients, evidence for the correspondence model was poor ($AIC_w \approx 0$). This means that their data was better explained by a simple vertical or horizontal line that indicates lack of retinotopic structure in one or both of the underlying maps, as expected given the lack of cone function in untreated ACHM and the low likelihood of finding evidence for cone-mediated map structure on these indices when no true map is present (Supplement 4).

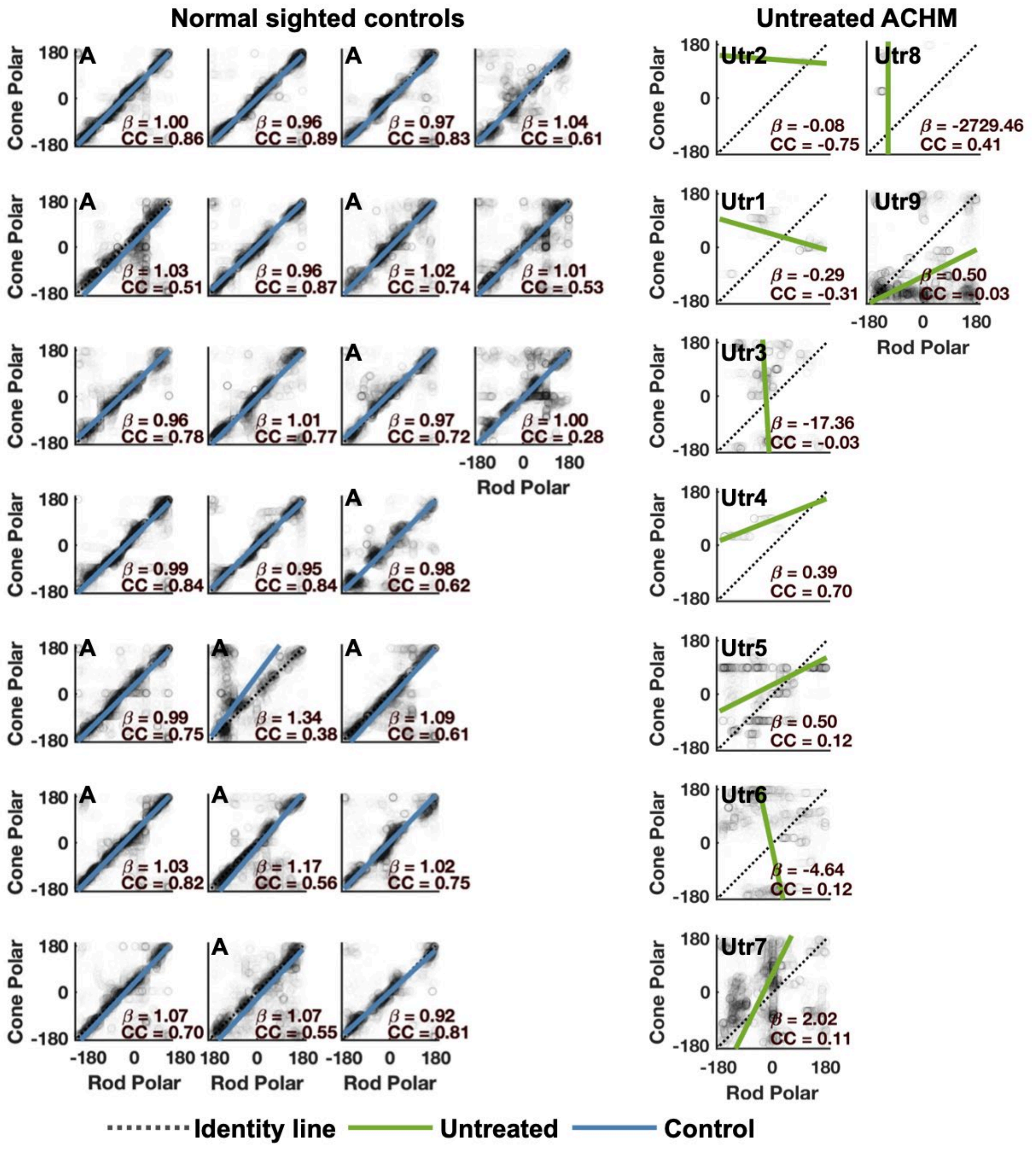


*Figure S6a. Cone-mediated retinotopic signal in visual cortex. Rod-mediated polar angle estimates from left and right V1-3 (x-axis) plotted against cone-mediated polar angle estimates (y-axis). The clustering of data around the identity line (dotted grey line) in normal sighted controls show the presentation of a cone map structure in these participants compared to the lack of cone-mediated signal in patients. Note that data from two normal sighted controls is not presented here since those participants were younger than 6 years and cone maps were not collected for them. In addition, data from two normal sighted participants whose data is shown in main figures as representative control comparisons is not presented here either. Solid lines indicate the orthogonal linear regression fit to these data for ACHM untreated patients (green) and normal sighted controls (light blue). β: slope of fitted line; CC: Fisher-Lee circular correlation coefficient.*

In Main Figure 2, we test for the cone-mediated signalling in visual cortex by comparing polar angle position estimates obtained with cone-selective pRF mapping to those obtained with rod-selective pRF mapping. Here we present the same analysis for the eccentricity position estimate, (Figure S6B) and for the X (horizontal) and Y (vertical) position parameters of the pRF

model that the polar angle and eccentricity position estimates are computed from (Figure S6C). As can be seen, the main finding of the emergence of a cone-mediated retinotopic map after treatment for patients in Tr1 and Tr2 but not patients Tr3 and Tr4 is broadly replicated for these alternative pRF position estimates. However, while for patient Tr2 all indices of a cone-mediated retinotopic map (linear regression slope, intercept, and correlation coefficient) have improved into the normal sighted range after the gene therapy, for patient Tr1 the Y (vertical) pRF position estimates and the eccentricity values show improved correspondence across the rod-mediated and cone-mediated map, but still fall outside the normal range and also show a lower than normal correlation. This is potentially consistent with the cortical visual field coverage plot of patient Tr1 (Main Figure 3), which shows that cone-mediated pRFs from V1-3 have stronger coverage in the upper visual field, encoded by the lower part of the retina.

**Table S6a: Participant details for eccentricity fMRI data measures regarding the correspondence and correlation between the cone- and rod-driven cortical maps.**

| | | | fMRI Eccentricity | | | |
|---|---|---|---|---|---|---|
| **Label** | **Age group** | **Genotype** | **Intercept** | **Slope** | **AIC$_w$[a]** | **Rho[b]** |
| **Pre-treatment treated patients with ACHM** | | | | | | |
| Tr1 | Child | CNGA3 | 2.17 | 0.04 | 0.00 | 0.13 |
| Tr2 | Child | CNGA3 | 1.58 | 0.18 | 0.00 | 0.18 |
| Tr3 | Child | CNGB3 | 1.73 | -0.02 | 0.00 | -0.15 |
| Tr4 | Child | CNGA3 | 24.94 | -3.80 | 0.00 | -0.28 |
| **Post-treatment treated patients with ACHM** | | | | | | |
| Tr1 | Child | CNGA3 | -4.89 | 1.98 | 0.95 | 0.18 |
| Tr2 | Child | CNGA3 | -0.55 | 0.91 | 1.00 | 0.67 |
| Tr3 | Child | CNGB3 | 2.71 | 0.08 | 0.00 | 0.18 |
| Tr4 | Child | CNGA3 | 1.07 | -0.03 | 0.00 | -0.22 |
| **Normal sighted controls** | | | | | | |
| C1 | Child | [c] | -0.99 | 1.22 | 1.00 | 0.79 |
| C2 | Child | [c] | -0.70 | 1.23 | 1.00 | 0.80 |

[a]Akaike Weight
[b]Pearson's correlation coefficient
[c]Normal sighted control with no achromatopsia

**Table S6b: Participant details for fMRI X and Y data measures regarding the correspondence and correlation between the cone- and rod-driven cortical maps**

| | | | fMRI X | | | | fMRI Y | | | |
|---|---|---|---|---|---|---|---|---|---|---|
| **Label** | **Age group** | **Genotype** | **Intercept** | **Slope** | **AIC$_w$[a]** | **Rho[b]** | **Intercept** | **Slope** | **AIC$_w$[a]** | **Rho[b]** |
| **Pre-treatment treated patients with ACHM** | | | | | | | | | | |
| Tr1 | Child | CNGA3 | -1.36 | -0.01 | 0.00 | -0.11 | 1.93 | 0.12 | 0.00 | 0.42 |
| Tr2 | Child | CNGA3 | 1.50 | -0.01 | 0.00 | -0.01 | 0.60 | 0.11 | 0.00 | 0.25 |
| Tr3 | Child | CNGB3 | 0.82 | 0.02 | 0.00 | 0.10 | 0.83 | 0.01 | 0.00 | 0.13 |
| Tr4 | Child | CNGA3 | 6.77 | -1.14 | 0.00 | -0.51 | 1.11 | 0.30 | 0.00 | 0.20 |
| **Post-treatment treated patients with ACHM** | | | | | | | | | | |
| Tr1 | Child | CNGA3 | -1.25 | 1.11 | 1.00 | 0.73 | 1.57 | 1.52 | 1.00 | 0.22 |
| Tr2 | Child | CNGA3 | 1.49 | 0.82 | 1.00 | 0.76 | -0.52 | 0.79 | 1.00 | 0.81 |
| Tr3 | Child | CNGB3 | 2.00 | 0.03 | 0.00 | 0.12 | 1.88 | -0.01 | 0.00 | -0.03 |
| Tr4 | Child | CNGA3 | 0.79 | -0.04 | 0.00 | -0.31 | -0.04 | -0.02 | 0.00 | -0.20 |

| | | | | | | | | | | |
|---|---|---|---|---|---|---|---|---|---|---|
| **Normal sighted controls** | | | | | | | | | | |
| C1 | Child | c | -0.32 | 1.00 | 1.00 | 0.90 | 0.19 | 1.18 | 1.00 | 0.87 |
| C2 | Child | c | -0.90 | 1.08 | 1.00 | 0.82 | -0.34 | 1.19 | 1.00 | 0.87 |

[a]Akaike Weight
[b]Pearson's correlation coefficient
[c]Normal sighted control with no achromatopsia

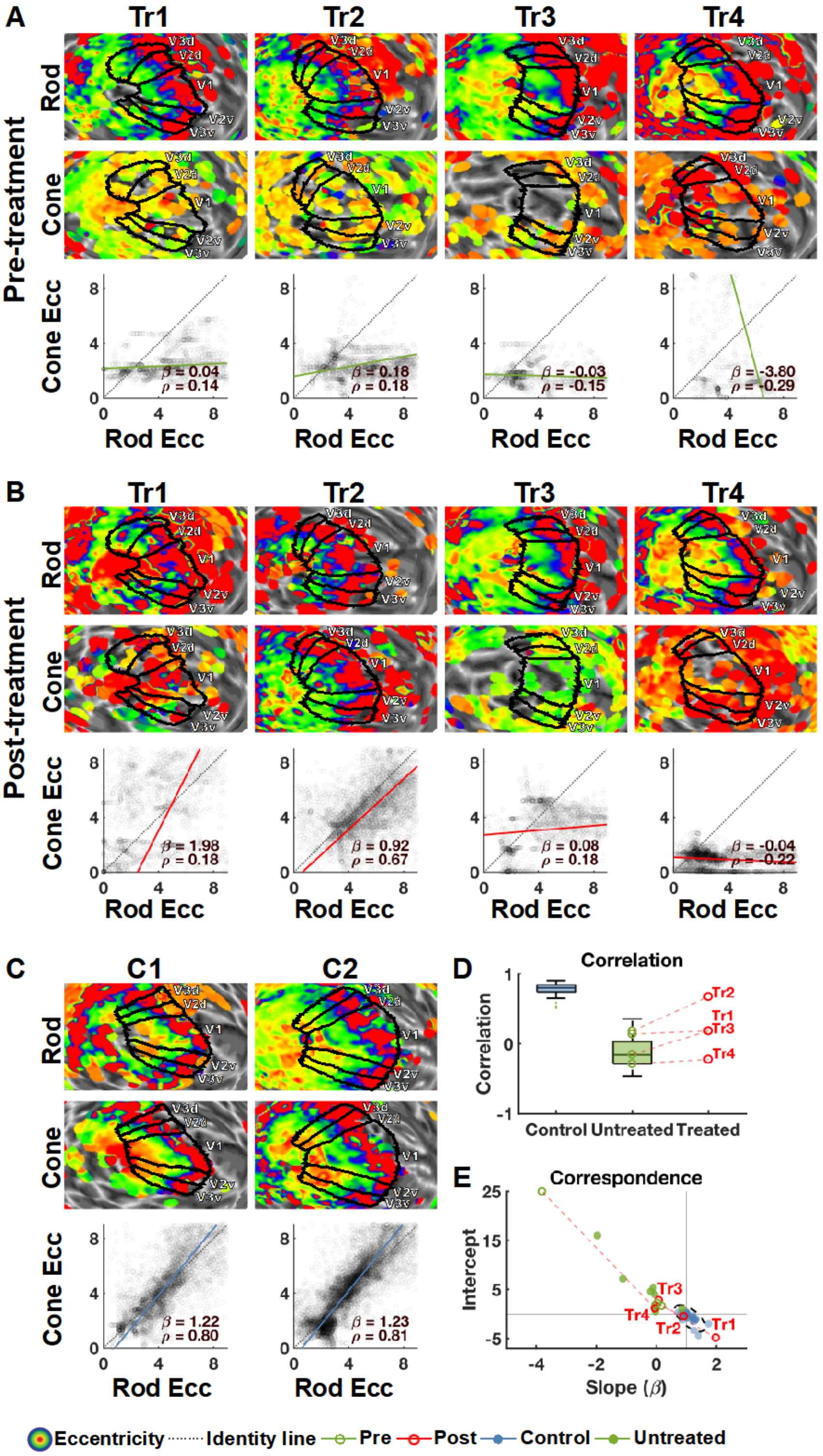


*Figure S6b. Cone-mediated versus rod-mediated eccentricity map organisation in areas V1-3 in 4 treated patients Tr1-4 before (A) and after (B) treatment, and in 2 age-matched normal sighted controls (C). For each measure, unthresholded rod- and cone mediated eccentricity estimates were projected onto the left hemisphere cortical surface,*

*inflated to a sphere and zoomed in on. V1-3 labels were drawn based on individual polar maps from an independent pRF condition that stimulated both rods and cones. To test for cone-mediated retinotopic signals in visual cortex, cone-mediated eccentricity values (y-axis) from left and right V1-3 are scattered against rod-mediated eccentricity values (x-axis). Dark blue dotted identity line (slope 1, intercept 0) indicates the predicted correspondence between rod- and cone-mediated pRF positions if a cone map is present. Solid lines indicate the orthogonal linear regression line fit to these data for ACHM patients pre-treatment (green), post-treatment (red), and normal sighted controls (light blue). β is the slope of this line. CC is the circular Fisher Lee correlation coefficient between rod and cone eccentricity values. D) Cone/rod map correlation: Box plots of circular correlations between rod and cone maps for normal sighted controls (blue), baseline patients with ACHM (green), and the 4 treated case study patients Tr1-4, with data shown pre- (green unfilled circles) and post-treatment (red unfilled circles). After treatment, correlation between the rod and cone-mediated eccentricity map has improved into the normal range in patient Tr2, whilst remaining lower in patients Tr1, Tr3 and Tr4. E) Cone/rod map correspondence: the slope and intercept for the linear regression line scattered against each other for the same groups as in D. Normal sighted control data clusters around β=1, and intercept=0, indicating high spatial rod/cone map correspondence. The dotted circle is the 95% range of these data. Data from all untreated baseline ACHM patients falls outside this range, in line with the low chance of observing this correspondence by chance (supplement 3). After treatment, regression slope measures from patients Tr1&Tr2 approach the normal sighted range, whilst those of Tr3&Tr4 do not - although only measures from patient Tr2 fall within the 95% range of measures seen in sighted controls.*

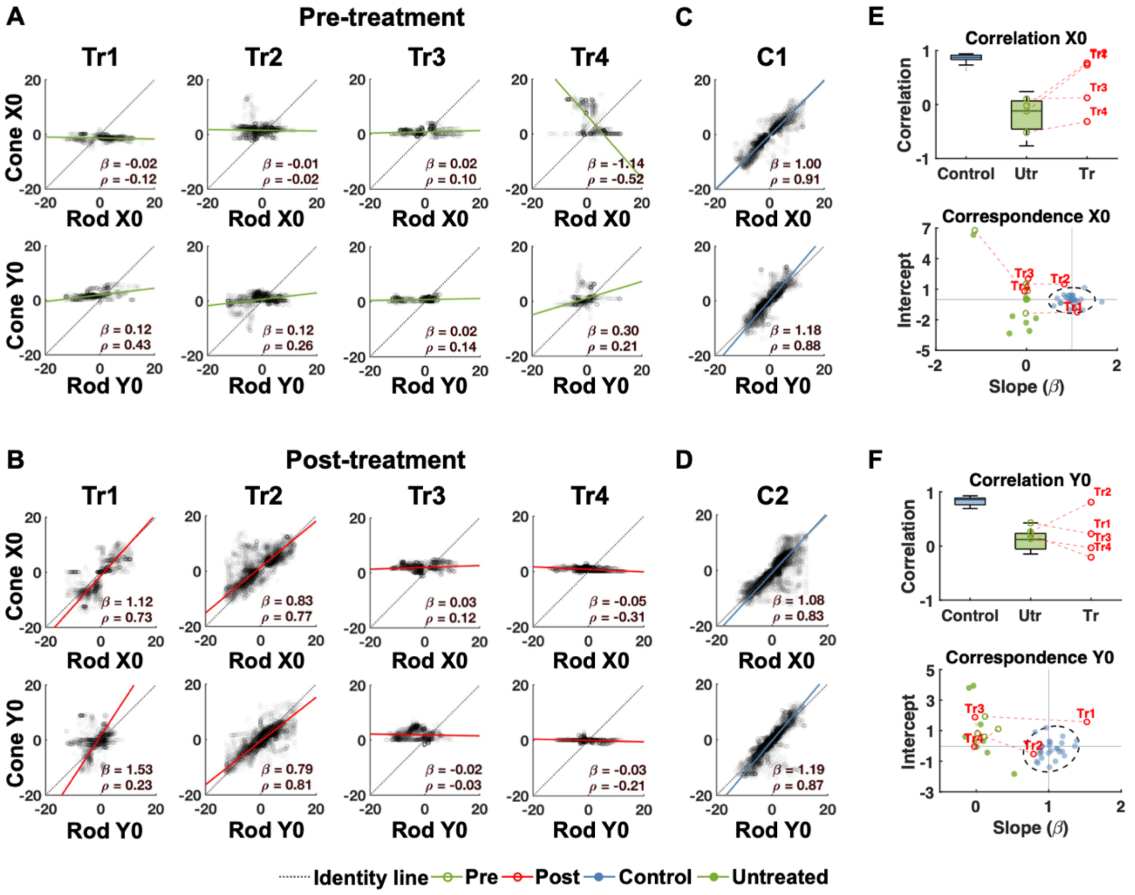


*Figure S6c. Same as in figure S6b but results are plotted for the rod and cone-mediated X and Y pRF position estimates instead of the eccentricity values. The X and Y pRF estimates are not displayed on cortical surface as these are typically converted to polar angle and eccentricity values for displaying, and these data are shown in Main Figure 2 and Figure S4b. (A) X and Y pRF position estimates from all 4 treated patients showed poor correspondence and*

*correlation across the rod and cone map before treatment. Panels C&D show that this was the case for all untreated patients with ACHM (13 in total). After treatment (B), correlation between the X position estimate from the rod and cone map (C&D) resembled those of age-matched controls and had improved into the normal range in for patients Tr1 and Tr2, whilst remaining lower in patients Tr3 and Tr4. For the Y position estimate, only patient Tr2 showed improved correlation between cone and rod-mediated estimates into the normal sighted. Measures of rod/cone map correspondence (C&E) (regression intercept and slope) had both shifted toward the normal sighted range after treatment for patients Tr1&Tr2, whilst those of Tr3&Tr4 had not. However, only for patient Tr2 did the improved correspondence between rod and cone map Y position estimates result in values within the 95% range of sighted controls.*

## Supplementary Materials 7: pRF sizes with non-selective stimuli

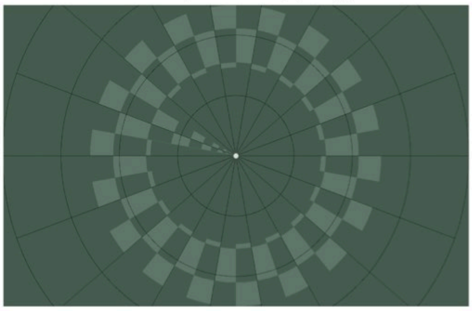


*Figure S7a: Non-selective pRF mapping stimulus. This ring/wedge stimulus was designed, using a silent substitution approach, to activate rods and L&M cones equally. It was presented in the scanner at a maximum luminance level of 0.5 cd/$m^2$.*

In a recent study, McKyton and colleagues (2021) reported atypically large (>4 degrees) rod-mediated pRF sizes in two adult patients with CNGA3-linked ACHM, in visual cortex areas V1-3, combined with an atypical eccentricity map. These patients also underwent gene therapy. After treatment, McKyton and colleagues reported a decrease in their pRF size, which they attributed to new cone function. pRF measures from these individuals were obtained under photopic conditions (max 180 cd/$m^2$) in which both rods and cones were activated, but rods were likely partially saturated. Eye-movements were not recorded online during scanning. With these stimuli it is therefore not possible to ascertain whether differences between patients and controls, nor within patients after treatment, reflect changes in the cone-system, rod-system, or fixational eye movements (especially those associated with nystagmus).

In our study, we addressed this problem by using pRF stimuli that activated rod and cone photoreceptors independently under appropriate lighting levels for each photoreceptor type (near scotopic for rods, low photopic for cones). Keeping light-levels low was crucial for testing children with ACHM, because due to photophobia the higher photopic light range is typically uncomfortable to look at and not tolerated well. These cone and rod-selective measurements allowed us, for the first time, to pinpoint functional change after gene therapy in ACHM to the specific photoreceptor system targeted by this treatment. It also allowed us to separately compare the pRF tuning estimates mediated by rods and recovered cones in ACHM to normal visual development. pRF mapping results for the rod-selective and cone-selective conditions are discussed in the main text. Here however, we describe results from a third pRF mapping condition that we collected, and which is best matched to the study by McKyton and colleagues.

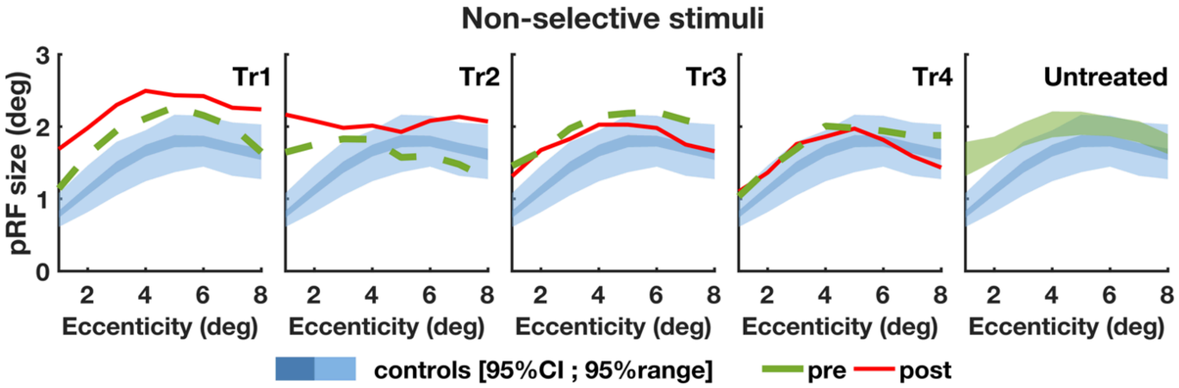


*Figure S7b. pRF size over eccentricity in the non-selective condition. Median pRF size for V1-3 across both hemispheres for each participant plotted against 1 degree eccentricity bins (range: 0.5-8.5). The 95% confidence interval (dark blue) and 95% range (light blue) of the normal-sighted control group (n=28) are plotted. The 95% confidence interval (green) of all 13 untreated baseline patients with ACHM (right) reveals ~1° larger pRF size estimates compared to normal sighted participants for small eccentricities. Pre-treatment data (dashed green line) and post-treatment data (solid red line) are plotted for treated patients Tr1-4. ecc: Eccentricity; deg: degrees; pre: pre-treatment; post: post-treatment; CI: confidence interval.*

In this condition, non-selective luminance-varying stimuli were used to activate both rod and cone photoreceptors (max luminance 0.5 $cd/m^2$). Figure S5b shows that we did not replicate McKyton et al.'s findings with these stimuli. Before treatment, we found that in the 4 treated patients - and indeed all untreated baseline patients with ACHM, pRF sizes were near the normal size, if slightly increased relative to controls around near-foveal eccentricities (by <1 degree difference). As described in the main text, pRF size estimates, especially near the fovea can appear larger as a result of fixation instability in ACHM (Clavagnier et al., 2015). It is therefore unclear to what extent the <1 degree difference in pRF size between normal sighted controls and patients with ACHM before treatment, arose from gaze differences, or the engagement of only the rod photoreceptor system in patients versus all photoreceptor types in healthy controls. One possible explanation for this discrepancy between the two studies is that the patients in Mckyton et al. were adults and here most of our baseline group were children, however we found no systematic age difference in pRF size: the mean pRF size across all eccentricities for the 2 adult patients in our sample were 1.39 deg and 1.35 deg, while the overall average of the group was 1.6 deg. This is in line with results from the rod-selective map: the mean rod-mediated pRF size across all eccentricities for the 2 adult patients were 1.66 deg and 1.42 deg, while the overall average of the group was 1.7 deg

We also did not replicate McKyton et al's result of a reduction in pRF size after gene therapy. If anything, in the two patients that showed clear treatment effects on our more direct measures of cortical cone function (Tr1, Tr2), pRFs were larger after treatment compared to their pre scan. Note that pRF size estimates are inherently more variable across subjects and sessions than pRF position parameters (van Dijk et al., 2016) and that fixation instability increases variability further (Clavagnier et al., 2015) so power to detect any small differences in pRF size due to new cone function are limited in our study.

# References


Clavagnier, S., Dumoulin, S. O., & Hess, R. F. (2015). Is the cortical deficit in amblyopia due to reduced cortical magnification, loss of neural resolution, or neural disorganization? *Journal of Neuroscience*, *35*(44), 14740–14755. https://doi.org/10.1523/JNEUROSCI.1101-15.2015

Dunn, M. J., Harris, C. M., Ennis, F. A., Margrain, T. H., Woodhouse, J. M., McIlreavy, L., & Erichsen, J. T. (2019). An automated segmentation approach to calibrating infantile nystagmus waveforms. *Behavior Research Methods*, *51*(5), 2074–2084. https://doi.org/10.3758/s13428-018-1178-5

Estévez, O., & Spekreijse, H. (1982). The "silent substitution" method in visual research. *Vision Research*, *22*(6), 681–691. https://doi.org/10.1016/0042-6989(82)90104-3

Rosengren, W., Nyström, M., Hammar, B., & Stridh, M. (2020). A robust method for calibration of eye tracking data recorded during nystagmus. *Behavior Research Methods*, *52*(1), 36–50. https://doi.org/10.3758/s13428-019-01199-0

Siegel, J. S., Power, J. D., Dubis, J. W., Vogel, A. C., Church, J. A., Schlaggar, B. L., & Petersen, S. E. (2014). Statistical improvements in functional magnetic resonance imaging analyses produced by censoring high-motion data points. *Human Brain Mapping*, *35*(5), 1981–1996. https://doi.org/10.1002/HBM.22307

Spitschan, M., & Woelders, T. (2018). The Method of Silent Substitution for Examining Melanopsin Contributions to Pupil Control. *Frontiers in Neurology*, *9*(NOV), 941. https://doi.org/10.3389/fneur.2018.00941

Stockman, A., & Sharpe, L. T. (2000). The spectral sensitivities of the middle- and long-wavelength-sensitive cones derived from measurements in observers of known genotype. *Vision Research*, *40*(13), 1711–1737. https://doi.org/10.1016/S0042-6989(00)00021-3

Tailor, V. K., Theodorou, M., Dahlmann-Noor, A. H., & Greenwood, J. A. (2020). The effect of eye movements on visual crowding in congenital nystagmus. *Journal of Vision*, *20*(11), 711. https://doi.org/10.1167/jov.20.11.711

van Dijk, J. A., de Haas, B., Moutsiana, C., & Schwarzkopf, D. S. (2016). Intersession reliability of population receptive field estimates. *NeuroImage*, *143*, 293–303. https://doi.org/10.1016/j.neuroimage.2016.09.013

Wyszecki, G., & Stiles, W. S. (1982). *Color science: concepts and methods, quantitative data and formulas*.
